# Thoracic and cardiovascular surgeries in Japan during 2020: Annual report by the Japanese Association for Thoracic Surgery

**DOI:** 10.1007/s11748-023-01979-8

**Published:** 2023-11-28

**Authors:** Committee for Scientific Affairs, The Japanese Association for Thoracic Surgery, Goro Matsumiya, Yukio Sato, Hiroya Takeuchi, Tomonobu Abe, Shunsuke Endo, Yasutaka Hirata, Michiko Ishida, Hisashi Iwata, Takashi Kamei, Nobuyoshi Kawaharada, Shunsuke Kawamoto, Kohji Kohno, Hiraku Kumamaru, Kenji Minatoya, Noboru Motomura, Rie Nakahara, Morihito Okada, Hisashi Saji, Aya Saito, Hideyuki Shimizu, Kenji Suzuki, Hirofumi Takemura, Tsuyoshi Taketani, Yasushi Toh, Wataru Tatsuishi, Hiroyuki Yamamoto, Takushi Yasuda, Masayuki Watanabe, Naoki Yoshimura, Masanori Tsuchida, Yoshiki Sawa

**Affiliations:** 1 The Japanese Association for Thoracic Surgery, Committee for Scientific Affairs, Tokyo, Japan; 2 Department of Cardiovascular Surgery, Chiba University Graduate School of Medicine, Chiba, Japan; 3 Department of Thoracic Surgery, University of Tsukuba, Tsukuba, Japan; 4 Department of Surgery, Hamamatsu University School of Medicine, Shizuoka, Japan; 5 Division of Cardiovascular Surgery, Department of General Surgical Science, Gunma University, Maebashi, Japan; 6 Thoracic Surgery, Jichi Medical University Saitama Medical Center, Omiya, Japan; 7 Department of Cardiac Surgery, The University of Tokyo Hospital, Tokyo, Japan; 8 Cardiac Surgery, Handa City Hospita, Aichi, Japan; 9 Department of General Thoracic Surgery, Gifu University Hospital, Gifu, Japan; 10 Department of Surgery, Graduate School of Medicine, Tohoku University, Sendai, Japan; 11 Department of Cardiovascular Surgery, Sapporo Medical University School of Medicine, Sapporo, Japan; 12 Department of Cardiovascular Surgery, Tohoku Medical and Pharmaceutical University Hospital, Sendai, Japan; 13 Department of Gastrointestinal Tract Surgery, Fukushima Medical University, Fukushima, Japan; 14 Department of Healthcare Quality Assessment, Graduate School of Medicine, The University of Tokyo, Tokyo, Kagoshima Japan; 15 Department of Cardiovascular Surgery, Graduate School of Medicine, Kyoto University, Kyoto, Japan; 16 Department of Cardiovascular Surgery, Toho University Sakura Medical Center, Chiba, Japan; 17 Division of Thoracic Surgery, Tochigi Cancer Center, Toshigi, Japan; 18 Surgical Oncology, Hiroshima University, Hiroshima, Japan; 19 Department of Chest Surgery, St. Marianna University School of Medicine, Kawasaki, Japan; 20 Department of Surgery, Yokohama City University, Graduate School of Medicine, Yokohama, Japan; 21 Department of Cardiovascular Surgery, Keio University, Tokyo, Japan; 22 Department of General Thoracic Surgery, Juntendo University School of Medicine, Tokyo, Japan; 23 Department of Cardiovascular Surgery, Kanazawa University, Kanazawa, Japan; 24 Department of Cardiovascular Surgery, Mitsui Memorial Hospital, Tokyo, Japan; 25 Department of Gastroenterological Surgery, National Hospital Organization Kyushu Cancer Center, Fukuoka, Japan; 26 Department of Surgery, Kindai University Faculty of Medicine, Osaka, Japan; 27 Department of Gastroenterological Surgery, Cancer Institute Hospital, Tokyo, Japan; 28 Department of Thoracic and Cardiovascular Surgery, University of Toyama, Graduate School of Medicine, Toyama, Japan; 29 Division of Thoracic and Cardiovascular Surgery, Niigata University Graduate School of Medical and Dental Sciences, Niigata, Japan; 30 Osaka University, Graduate School of Medicine, Osaka Police Hospital, Osaka, Japan

**Keywords:** Japan Cardiovascular Surgery Database (JCVSD), National Clinical Database (NCD), Annual report in Japan, Cardiovascular surgery, General thoracic surgery, Esophageal surgery

Since 1986, the Japanese Association for Thoracic Surgery has conducted annual thoracic surgery surveys throughout Japan to determine statistics on the number of procedures performed by surgical categories. Herein, we summarize the results of the association’s annual thoracic surgery surveys in 2020. We regret that, for various reasons, this report has been delayed to 2023.

Adhering to the norm thus far, thoracic surgery had been classified into three categories, including cardiovascular, general thoracic, and esophageal surgeries, with patient data for each group being examined and analyzed. We honor and value all members’ continued professional support and contributions.

Incidence of hospital mortality was included in the survey to determine nationwide status, which has contributed to Japanese surgeons’ understanding of the present status of thoracic surgery in Japan while helping in surgical outcome improvements by enabling comparisons between their work and that of others. This approach has enabled the association to gain a better understanding of present problems and prospects, which is reflected in its activities and member education.

The 30-day mortality (also known as *operative mortality*) is defined as death within 30 days of surgery, regardless of the patient’s geographic location, including post-discharge from the hospital. *Hospital mortality* is defined as death within any time interval following surgery among patients yet to be discharged from the hospital.

Transfer to a nursing home or a rehabilitation unit is considered hospital discharge unless the patient subsequently dies of complications from surgery, while hospital-to-hospital transfer during esophageal surgery is not considered a form of discharge. In contrast, hospital-to-hospital transfer 30 days following cardiovascular and general thoracic surgeries are considered discharge given that National Clinical Database (NCD)-related data were used in these categories.

Severe Acute Respiratory Syndrpme Coronavirus-2 (SARS-CoV-2), the causative pathogen for the coronavirus disease 2019 (COVID-19), first emerged in Wuhan, China, in December 2019 and by March 2020, it was declared a pandemic [1]. The pandemic of SARS-CoV-2 resulted in a global healthcare and financial crisis. There was a significant estimated reduction in national case volume of cardiac surgeries and the cumulative backlog of patients in the United State [2]. We have to estimate the nationwide effect of SARS-CoV-2 pandemic on cardiovascular, general thoracic, and esophageal surgeries in Japan, with surgical volume, outcomes and patient data for each group.

## Survey abstract

All data on cardiovascular, general thoracic, and esophageal surgeries were obtained from the NCD. In 2018, the data collection method for general thoracic and esophageal surgeries had been modified from self-reports using questionnaire sheets following each institution belonging to the Japanese Association for Thoracic Surgery to an automatic package downloaded from the NCD in Japan.

The data collection related to cardiovascular surgery (initially self-reported using questionnaire sheets in each participating institution up to 2014) changed to downloading an automatic package from the Japanese Cardiovascular Surgery Database (JCVSD), which is a cardiovascular subsection of the NCD in 2015.

## Final report: 2020

### (A) Cardiovascular surgery

We are extremely pleased with the cooperation of our colleagues (members) in completing the cardiovascular surgery survey, which has undoubtedly improved the quality of this annual report. We are truly grateful for the significant efforts made by all participants within each participating institution in completing the JCVSD/NCD.

**Fig. 1:**
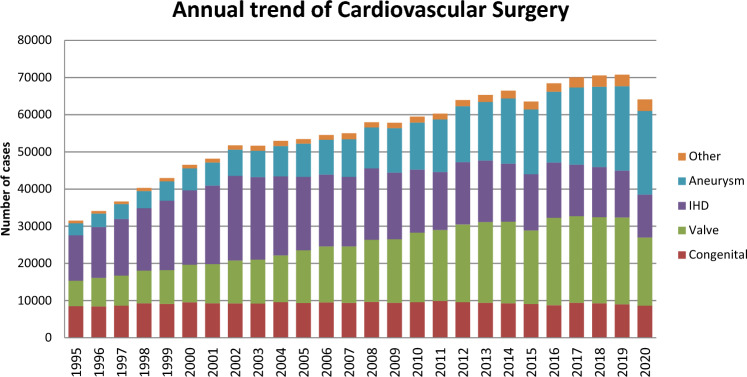
Annual trend of cardiovascular surgery

Figure 1 illustrates the development of cardiovascular surgery in Japan over the past 34 years. Aneurysm surgery includes only surgeries for thoracic and thoracoabdominal aortic aneurysms. Extra-anatomic bypass surgery for thoracic aneurysm and pacemaker implantation have been excluded from the survey since 2015. Assist device implantations were not included in the total number of surgical procedures but were included in the survey.

A total of 64,075 cardiovascular surgeries, including 54 heart transplants, had been performed in 2020, with a 9.5% decrease compared to that in 2019 (*n* = 70,769). For the first time since the beginning of data collection, except for the year 2015 when there was a decrease due to a change in data collection and aggregation methods, a decline in the number of cases has been observed. Although the impact of the COVID-19 pandemic is suggested, this will be reported separately.

Compared to data for 2019 [3] and 2010 [4], data for 2020 showed 4.6% (8595 vs. 9006) and 10.1% fewer surgeries for congenital heart disease, 21.3% (18,366 vs. 23,340) fewer and 1.9% fewer surgeries for valvular heart disease, 8.6% (11,524 vs. 12,603) and 31.9% fewer surgeries for ischemic heart procedures, and 0.7% (22,540 vs. 22,708) fewer and 77.3% more surgeries for thoracic aortic aneurysm, respectively. Data for individual categories are summarized in Tables 1, 2, 3, 4, 5, 6.

**Table 1:** Congenital (total; 8595) (1) CPB (+) (total; 6543)

	Neonate				Infant				1–17 years				≥ 18 years				Total			
	Cases	30-Day mortality		Hospital mortality	Cases	30-Day mortality		Hospital mortality	Cases	30-Day mortality		Hospital mortality	Cases	30-Day mortality		Hospital mortality	Cases	30-Day mortality		Hospital mortality
		Hospital	After discharge			Hospital	After discharge			Hospital	After discharge			Hospital	After discharge			Hospital	After discharge	
PDA	2	0	0	0	4	0	0	0	2	0	0	1 (50.0)	16	0	0	0	24	0	0	1 (4.2)
Coarctation (simple)	10	0	0	0	13	0	0	0	9	0	0	0	15	0	0	0	47	0	0	0
+ VSD	57	1 (1.8)	0	3 (5.3)	45	1 (2.2)	0	2 (4.4)	19	0	0	0	2	0	0	0	123	2 (1.6)	0	5 (4.1)
+ DORV	4	0	0	0	3	0	0	0	2	0	0	0	0	0	0	0	9	0	0	0
+ AVSD	5	0	0	0	7	0	0	0	1	0	0	0	0	0	0	0	13	0	0	0
+ TGA	2	0	0	0	3	0	0	0	2	0	0	0	0	0	0	0	7	0	0	0
+ SV	0	0	0	0	0	0	0	0	0	0	0	0	0	0	0	0	0	0	0	0
+ Others	7	0	0	0	8	0	0	1 (12.5)	4	0	0	1 (25.0)	1	0	0	0	20	0	0	2 (10.0)
Interrupt. of Ao (simple)	0	0	0	0	0	0	0	0	0	0	0	0	0	0	0	0	0	0	0	0
+ VSD	13	0	0	0	19	1 (5.3)	0	1 (5.3)	17	0	0	0	0	0	0	0	49	1 (2.0)	0	1 (2.0)
+ DORV	1	0	0	0	1	0	0	0	0	0	0	0	0	0	0	0	2	0	0	0
+ Truncus	1	0	0	0	5	1 (20.0)	0	1 (20.0)	3	1 (33.3)	0	1 (33.3)	0	0	0	0	9	2 (22.2)	0	2 (22.2)
+ TGA	0	0	0	0	0	0	0	0	0	0	0	0	0	0	0	0	0	0	0	0
+ Others	3	0	0	0	1	0	0	0	3	0	0	0	0	0	0	0	7	0	0	0
Vascular ring	0	0	0	0	1	0	0	0	1	0	0	0	0	0	0	0	2	0	0	0
PS	2	0	0	0	23	0	0	0	78	0	0	0	17	0	0	0	120	0	0	0
PA·IVS or critical PS	15	0	0	0	56	1 (1.8)	0	1 (1.8)	57	0	0	2 (3.5)	5	0	0	0	133	1 (0.8)	0	3 (2.3)
TAPVR	109	2 (1.8)	0	8 (7.3)	52	2 (3.8)	0	3 (5.8)	17	0	0	0	2	0	0	0	180	4 (2.2)	0	11 (6.1)
PAPVR ± ASD	0	0	0	0	3	0	0	0	43	0	0	0	9	0	0	0	55	0	0	0
ASD	1	0	0	0	40	0	0	0	398	0	0	0	749	8 (1.1)	0	8 (1.1)	1188	8 (0.7)	0	8 (0.7)
Cor triatriatum	2	0	0	0	6	0	0	0	5	0	0	0	0	0	0	0	13	0	0	0
AVSD (partial)	3	1 (33.3)	0	1 (33.3)	8	0	0	0	34	0	0	0	9	0	0	0	54	1 (1.9)	0	1 (1.9)
AVSD (complete)	6	0	0	1 (16.7)	101	1 (1.0)	0	1 (1.0)	115	1 (0.9)	0	3 (2.6)	4	0	0	0	226	2 (0.9)	0	5 (2.2)
+ TOF or DORV	0	0	0	0	6	0	0	0	10	0	0	0	0	0	0	0	16	0	0	0
+ Others	0	0	0	0	0	0	0	0	0	0	0	0	0	0	0	0	0	0	0	0
VSD (subarterial)	2	0	0	0	98	0	0	0	144	0	0	0	9	0	0	0	253	0	0	0
VSD (perimemb./muscular)	13	0	0	0	675	1 (0.1)	0	2 (0.3)	329	0	0	0	26	1 (3.8)	0	1 (3.8)	1043	2 (0.2)	0	3 (0.3)
VSD (type unknown)	0	0	0	0	0	0	0	0	0	0	0	0	105	1 (1.0)	0	1 (1.0)	105	1 (1.0)	0	1 (1.0)
VSD + PS	0	0	0	0	33	0	0	0	16	0	0	0	2	1 (50.0)	0	1 (50.0)	51	1 (2.0)	0	1 (2.0)
DCRV ± VSD	0	0	0	0	8	0	0	0	27	0	0	0	8	0	0	0	43	0	0	0
Aneurysm of sinus of Valsalva	0	0	0	0	1	0	0	0	1	0	0	0	2	0	0	0	4	0	0	0
TOF	7	0	0	0	156	0	0	1 (0.6)	156	0	0	0	45	1 (2.2)	0	2 (4.4)	364	1 (0.3)	0	3 (0.8)
PA + VSD	8	0	0	1 (12.5)	76	2 (2.6)	0	3 (3.9)	108	0	0	0	11	0	0	0	203	2 (1.0)	0	4 (2.0)
DORV	14	0	0	0	111	1 (0.9)	0	3 (2.7)	160	2 (1.3)	0	2 (1.3)	7	0	0	0	292	3 (1.0)	0	5 (1.7)
TGA (simple)	89	0	1 (1.1)	0	5	0	0	0	5	0	0	0	3	0	0	0	102	0	1 (1.0)	0
+ VSD	32	0	0	0	12	0	0	0	6	0	0	0	1	0	0	0	51	0	0	0
VSD + PS	0	0	0	0	33	0	0	0	16	0	0	0	2	1 (50.0)	0	1 (50.0)	51	1 (2.0)	0	1 (2.0)
Corrected TGA	3	0	0	0	13	0	0	0	26	0	0	0	11	0	0	0	53	0	0	0
Truncus arteriosus	11	1 (9.1)	0	1 (9.1)	18	0	0	0	23	0	0	0	4	0	0	0	56	1 (1.8)	0	1 (1.8)
SV	30	4 (13.3)	0	10 (33.3)	167	7 (4.2)	0	12 (7.2)	176	1 (0.6)	0	1 (0.6)	20	0	0	0	393	12 (3.1)	0	23 (5.9)
TA	3	0	0	0	30	1 (3.3)	0	1 (3.3)	49	0	0	0	2	0	0	0	84	1 (1.2)	0	1 (1.2)
HLHS	38	4 (10.5)	0	13 (34.2)	119	5 (4.2)	0	7 (5.9)	78	2 (2.6)	0	2 (2.6)	1	0	0	0	236	11 (4.7)	0	22 (9.3)
Aortic valve lesion	3	0	0	0	13	0	0	0	102	0	0	1 (1.0)	29	0	0	0	147	0	0	1 (0.7)
Mitral valve lesion	1	0	0	0	37	1 (2.7)	0	1 (2.7)	64	0	0	1 (1.6)	24	0	0	2 (8.3)	126	1 (0.8)	0	4 (3.2)
Ebstein	8	1 (12.5)	0	2 (25.0)	13	0	0	0	30	0	0	1 (3.3)	7	0	0	0	58	1 (1.7)	0	3 (5.2)
Coronary disease	0	0	0	0	6	0	0	0	19	0	0	0	7	0	0	0	32	0	0	0
Others	7	1 (14.3)	0	1 (14.3)	14	0	0	0	37	0	0	1 (2.7)	211	2 (0.9)	0	2 (0.9)	269	3 (1.1)	0	4 (1.5)
Conduit failure	1	0	0	0	0	0	0	0	12	0	0	0	6	0	0	0	19	0	0	0
Redo (excluding conduit failure)	2	1 (50.0)	0	1 (50.0)	50	3 (6.0)	0	4 (8.0)	71	2 (2.8)	0	3 (4.2)	88	3 (3.4)	0	4 (4.5)	211	9 (4.3)	0	12 (5.7)
Total	515	16 (3.1)	1 (0.2)	42 (8.2)	2093	28 (1.3)	0	44 (2.1)	2475	9 (0.4)	0	20 (0.8)	1460	18 (1.2)	0	22 (1.5)	6543	71 (1.1)	1 (0.0)	128 (2.0)
(), % mortality																				
*CPB* cardiopulmonary bypass, *PDA* patent ductus arteriosus, *VSD* ventricular septal defect, *DORV* double outlet right ventricle, *AVSD* atrioventricular septal defect, *TGA* transposition of great arteries, *SV* single ventricle, *Interrupt. of Ao.* interruption of aorta, *PS* pulmonary stenosis, *PA-IVS* pulmonary atresia with intact ventricular septum, *TAPVR* total anomalous pulmonary venous return, *PAPVR* partial anomalous pulmonary venous return, *ASD* atrial septal defect, *TOF* tetralogy of Fallot, *DCRV* double-chambered right ventricle, *TA* tricuspid atresia, *HLHS* hypoplastic left heart syndrome, *RV-PA* right ventricle-pulmonary artery																				

(2) CPB (−) (total; 2052)																				
	Neonate				Infant				1–17 years				≥ 18 years				Total			
	Cases	30-Day mortality		Hospital mortality	Cases	30-Day mortality		Hospital mortality	Cases	30-Day mortality		Hospital mortality	Cases	30-Day mortality		Hospital mortality	Cases	30-Day mortality		Hospital mortality
		Hospital	After discharge			Hospital	After discharge			Hospital	After discharge			Hospital	After discharge			Hospital	After discharge	
PDA	291	7 (2.4)	0	14 (4.8)	115	0	0	1 (0.9)	14	0	0	0	0	0	0	0	420	7 (1.7)	0	15 (3.6)
Coarctation (simple)	11	0	0	0	10	0	0	0	5	0	0	0	1	0	0	0	27	0	0	0
+ VSD	44	0	0	3 (6.8)	19	1 (5.3)	0	2 (10.5)	0	0	0	0	0	0	0	0	63	1 (1.6)	0	5 (7.9)
+ DORV	4	0	0	0	0	0	0	0	0	0	0	0	0	0	0	0	4	0	0	0
+ AVSD	2	0	0	0	3	0	0	1 (33.3)	0	0	0	0	0	0	0	0	5	0	0	1 (20.0)
+ TGA	3	0	0	0	0	0	0	0	0	0	0	0	0	0	0	0	3	0	0	0
+ SV	0	0	0	0	0	0	0	0	0	0	0	0	0	0	0	0	0	0	0	0
+ Others	7	1 (14.3)	0	1 (14.3)	4	0	0	0	3	0	0	0	0	0	0	0	14	1 (7.1)	0	1 (7.1)
Interrupt. of Ao (simple)	0	0	0	0	0	0	0	0	0	0	0	0	0	0	0	0	0	0	0	0
+ VSD	17	0	0	0	11	1 (9.1)	0	1 (9.1)	1	0	0	0	0	0	0	0	29	1 (3.4)	0	1 (3.4)
+ DORV	0	0	0	0	0	0	0	0	0	0	0	0	0	0	0	0	0	0	0	0
+ Truncus	5	0	0	0	1	1 (100.0)	0	1 (100.0)	0	0	0	0	0	0	0	0	6	1 (16.7)	0	1 (16.7)
+ TGA	0	0	0	0	0	0	0	0	0	0	0	0	0	0	0	0	0	0	0	0
+ Others	3	0	0	0	2	0	0	0	0	0	0	0	0	0	0	0	5	0	0	0
Vascular ring	4	0	0	0	11	0	0	0	5	0	0	0	0	0	0	0	20	0	0	0
PS	2	0	0	0	5	0	0	1 (20.0)	1	0	0	0	0	0	0	0	8	0	0	1 (12.5)
PA·IVS or critical PS	12	2 (16.7)	0	2 (16.7)	23	1 (4.3)	0	1 (4.3)	6	1 (16.7)	0	1 (16.7)	1	0	0	0	42	4 (9.5)	0	4 (9.5)
TAPVR	24	2 (8.3)	0	3 (12.5)	13	1 (7.7)	0	2 (15.4)	0	0	0	0	0	0	0	0	37	3 (8.1)	0	5 (13.5)
PAPVR ± ASD	0	0	0	0	1	0	0	0	0	0	0	0	0	0	0	0	1	0	0	0
ASD	2	0	0	0	1	0	0	0	2	0	0	0	1	1 (100.0)	0	1 (100.0)	6	1 (16.7)	0	1 (16.7)
Cor triatriatum	0	0	0	0	0	0	0	0	0	0	0	0	0	0	0	0	0	0	0	0
AVSD (partial)	3	1 (33.3)	0	1 (33.3)	1	0	0	0	0	0	0	0	0	0	0	0	4	1 (25.0)	0	1 (25.0)
AVSD (complete)	51	1 (2.0)	0	2 (3.9)	75	2 (2.7)	0	2 (2.7)	9	1 (11.1)	0	1 (11.1)	0	0	0	0	135	4 (3.0)	0	5 (3.7)
+ TOF or DORV	0	0	0	0	1	0	0	0	1	0	0	0	1	0	0	0	3	0	0	0
+ Others	0	0	0	0	0	0	0	0	0	0	0	0	0	0	0	0	0	0	0	0
VSD (subarterial)	3	0	0	1 (33.3)	8	0	0	0	1	0	0	0	0	0	0	0	12	0	0	1 (8.3)
VSD (perimemb./muscular)	55	1 (1.8)	0	1 (1.8)	148	2 (1.4)	0	2 (1.4)	2	0	0	0	3	1 (33.3)	0	1 (33.3)	208	4 (1.9)	0	4 (1.9)
VSD (type unknown)	0	0	0	0	0		0	0	0	0	0	0	1	0	0	0	1	0	0	0
VSD + PS	0	0	0	0	1	0	0	0	0	0	0	0	0	0	0	0	1	0	0	0
DCRV ± VSD	0	0	0	0	0	0	0	0	0	0	0	0	0	0	0	0	0	0	0	0
Aneurysm of sinus of Valsalva	0	0	0	0	0	0	0	0	0	0	0	0	0	0	0	0	0	0	0	0
TOF	13	0	0	0	49	0	0	0	5	0	0	0	1	0	0	0	68	0	0	0
PA + VSD	7	0	0	0	44	1 (2.3)	0	1 (2.3)	17	0	0	0	0	0	0	0	68	1 (1.5)	0	1 (1.5)
DORV	54	2 (3.7)	0	5 (9.3)	56	1 (1.8)	0	2 (3.6)	11	0	0	0	1	1 (100.0)	0	1 (100.0)	122	4 (3.3)	0	8 (6.6)
TGA (simple)	11	0	0	0	2	0	0	0	0	0	0	0	1	0	0	0	14	0	0	0
+ VSD	9	1 (11.1)	0	1 (11.1)	4	0	0	0	1	0	0	0	0	0	0	0	14	1 (7.1)	0	1 (7.1)
VSD + PS	0	0	0	0	0	0	0	0	0	0	0	0	0	0	0	0	0	0	0	0
Corrected TGA	17	0	0	0	9	0	0	0	13	0	0	0	1	0	0	0	40	0	0	0
Truncus arteriosus	19	1 (5.3)	0	1 (5.3)	12	0	0	0	2	0	0	0	1	0	0	0	34	1 (2.9)	0	1 (2.9)
SV	47	1 (2.1)	0	5 (10.6)	57	3 (5.3)	0	4 (7.0)	16	0	0	0	4	0	0	1 (25.0)	124	4 (3.2)	0	10 (8.1)
TA	17	0	0	2 (11.8)	16	0	0	1 (6.3)	2	0	0	0	1	0	0	0	36	0	0	3 (8.3)
HLHS	76	2 (2.6)	0	12 (15.8)	26	2 (7.7)	0	2 (7.7)	12	1 (8.3)	0	1 (8.3)	0	0	0	0	114	5 (4.4)	0	15 (13.2)
Aortic valve lesion	6	1 (16.7)	0	1 (16.7)	2	0	0	0	4	0	0	0	1	0	0	0	13	1 (7.7)	0	1 (7.7)
Mitral valve lesion	1	0	0	0	5	1 (20.0)	0	1 (20.0)	0	0	0	0	0	0	0	0	6	1 (16.7)	0	1 (16.7)
Ebstein	5	1 (20.0)	0	1 (20.0)	1	0	0	0	1	0	0	0	0	0	0	0	7	1 (14.3)	0	1 (14.3)
Coronary disease	0	0	0	0	0	0	0	0	3	0	0	0	0	0	0	0	3	0	0	0
Others	6	0	0	1 (16.7)	13	2 (15.4)	0	2 (15.4)	10	0	0	0	4	0	0	0	33	2 (6.1)	0	3 (9.1)
Conduit failure	0	0	0	0	1	0	0	0	1	0	0	0	0	0	0	0	2	0	0	0
Redo (excluding conduit failure)	36	1 (2.8)	0	5 (13.9)	118	9 (7.6)	0	19 (16.1)	120	2 (1.7)	0	4 (3.3)	26	4 (15.4)	0	5 (19.2)	300	16 (5.3)	0	33 (11.0)
Total	867	25 (2.9)	0	62 (7.2)	868	28 (3.2)	0	46 (5.3)	268	5 (1.9)	0	7 (2.6)	49	7 (14.3)	0	9 (18.4)	2052	65 (3.2)	0	124 (6.0)
(), % mortality																				
*CPB* cardiopulmonary bypass, *PDA* patent ductus arteriosus, *VSD* ventricular septal defect, *DORV* double outlet right ventricle, *AVSD* atrioventricular septal defect, *TGA* transposition of the great arteries, *SV* single ventricle, *Interrupt. of Ao.* interruption of aorta, *PS* pulmonary stenosis, *PA-IVS* pulmonary atresia with intact ventricular septum, *TAPVR* total anomalous pulmonary venous return, *PAPVR* partial anomalous pulmonary venous return, *ASD* atrial septal defect, *TOF* tetralogy of Fallot, *DCRV* double-chambered right ventricle, *TA* tricuspid atresia, *HLHS* hypoplastic left heart syndrome, *RV-PA* right ventricle-pulmonary artery																				

(3) Main procedure																					
		Neonate				Infant				1–17 years				≥ 18 years				Total			
		Cases	30-Day mortality		Hospital mortality	Cases	30-Day mortality		Hospital mortality	Cases	30-Day mortality		Hospital mortality	Cases	30-Day mortality		Hospital mortality	Cases	30-Day mortality		Hospital mortality
			Hospital	After discharge			Hospital	After discharge			Hospital	After discharge			Hospital	After discharge			Hospital	After discharge	
1	SP Shunt	118	3 (2.5)	0	7 (5.9)	310	3 (1.0)	0	7 (2.3)	32	0	0	0	2	0	0	0	462	6 (1.3)	0	14 (3.0)
2	PAB	266	8 (3.0)	0	17 (6.4)	304	6 (2.0)	0	8 (2.6)	9	0	0	0	0	0	0	0	579	14 (2.4)	0	25 (4.3)
3	Bidirectional Glenn or hemi-Fontan ± α	0	0	0	0	230	2 (0.9)	0	3 (1.3)	105	1 (1.0)	0	1 (1.0)	0	0	0	0	335	3 (0.9)	0	4 (1.2)
4	Damus-Kaye-Stansel operation	1	0	0	0	25	0	0	0	8	0	0	0	1	0	0	0	35	0	0	0
5	PA reconstruction/repair (including redo)	16	2 (12.5)	0	2 (12.5)	161	5 (3.1)	0	6 (3.7)	196	0	0	0	11	0	0	0	384	7 (1.8)	0	8 (2.1)
6	RVOT reconstruction/repair	4	0	0	0	207	1 (0.5)	0	2 (1.0)	267	0	0	0	42	0	0	0	520	1 (0.2)	0	2 (0.4)
7	Rastelli procedure	2	0	0	0	41	0	0	0	101	0	0	0	2	0	0	0	146	0	0	0
8	Arterial switch procedure	129	0	1 (0.8)	0	24	0	0	0	2	0	0	0	1	0	0	1 (100.0)	156	0	1 (0.6)	1 (0.6)
9	Atrial switch procedure	0	0	0	0	1	0	0	0	3	0	0	0	1	0	0	0	5	0	0	0
10	Double switch procedure	0	0	0	0	0	0	0	0	7	0	0	0	0	0	0	0	7	0	0	0
11	Repair of anomalous origin of CA	0	0	0	0	6	0	0	0	3	0	0	0	1	0	0	0	10	0	0	0
12	Closure of coronary AV fistula	0	0	0	0	0	0	0	0	4	0	0	0	1	0	0	0	5	0	0	0
13	Fontan/TCPC	0	0	0	0	0	0	0	0	353	1 (0.3)	0	3 (0.8)	25	0	0	0	378	1 (0.3)	0	3 (0.8)
14	Norwood procedure	28	1 (3.6)	0	9 (32.1)	84	7 (8.3)	0	10 (11.9)	5	0	0	0	0	0	0	0	117	8 (6.8)	0	19 (16.2)
15	Ventricular septation	0	0	0	0	0	0	0	0	0	0	0	0	0	0	0	0	0	0	0	0
16	Left side AV valve repair (including Redo)	2	1 (50.0)	0	1 (50.0)	40	1 (2.5)	0	2 (5.0)	56	0	0	0	26	1 (3.8)	0	1 (3.8)	124	3 (2.4)	0	4 (3.2)
17	Left side AV valve replace (including Redo)	0	0	0	0	9	1 (11.1)	0	1 (11.1)	36	0	0	2 (5.6)	23	0	0	2 (8.7)	68	1 (1.5)	0	5 (7.4)
18	Right side AV valve repair (including Redo)	14	2 (14.3)	0	3 (21.4)	91	1 (1.1)	0	1 (1.1)	83	2 (2.4)	0	2 (2.4)	58	0	0	1 (1.7)	246	5 (2.0)	0	7 (2.8)
19	Right side AV valve replace (including Redo)	0	0	0	0	6	1 (16.7)	0	1 (16.7)	10	0	0	2 (20.0)	35	0	0	1 (2.9)	51	1 (2.0)	0	4 (7.8)
20	Common AV valve repair (including Redo)	8	0	0	2 (25.0)	26	2 (7.7)	0	3 (11.5)	16	0	0	0	0	0	0	0	50	2 (4.0)	0	5 (10.0)
21	Common AV valve replace (including Redo)	2	0	0	0	6	1 (16.7)	0	2 (33.3)	4	1 (25.0)	0	1 (25.0)	2	0	0	0	14	2 (14.3)	0	3 (21.4)
22	Repair of supra-aortic stenosis	0	0	0	0	9	0	0	0	19	0	0	0	0	0	0	0	28	0	0	0
23	Repair of subaortic stenosis (including Redo)	3	0	0	0	3	0	0	0	32	0	0	0	3	1 (33.3)	0	1 (33.3)	41	1 (2.4)	0	1 (2.4)
24	Aortic valve plasty ± VSD Closure	0	0	0	0	10	0	0	0	44	1 (2.3)	0	1 (2.3)	2	0	0	0	56	1 (1.8)	0	1 (1.8)
25	Aortic valve replacement	0	0	0	0	2	0	0	0	27	0	0	0	31	0	0	0	60	0	0	0
26	AVR with annular enlargement	0	0	0	0	0	0	0	0	16	0	0	1 (6.3)	4	0	0	0	20	0	0	1 (5.0)
27	Aortic root Replace (except Ross)	0	0	0	0	0	0	0	0	7	0	0	0	16	1 (6.3)	0	2 (12.5)	23	1 (4.3)	0	2 (8.7)
28	Ross procedure	0	0	0	0	3	0	0	0	13	0	0	0					16	0	0	0
29	Bilateral pulmonary artery banding	166	6 (3.6)	0	24 (14.5)	15	0	0	2 (13.3)	1	0	0	0	0	0	0	0	182	6 (3.3)	0	26 (14.3)
Total		759	23 (3.0)	1 (0.1)	65 (8.6)	1613	31 (1.9)	0	48 (3.0)	1459	6 (0.4)	0	13 (0.9)	287	3 (1.0)	0	9 (3.1)	4118	63 (1.5)	1 (0.02)	135 (3.3)

(), % mortality

*SP* systemic-pulmonary, *PAB* pulmonary artery banding, *PA* pulmonary artery, *RVOT* right ventricular outflow tract, *CA* coronary artery, *AV fistula* arteriovenous fistula, *TCPC* total cavopulmonary connection, *AV valve* atrioventricular valve, *VSD* ventricular septal defect, *AVR* aortic valve replacement

**Table 2:** Acquired (total, (1) + (2) + (4) + (5) + (6) + (7) + isolated operations for arrhythmia in (3); 32,509

(1) Valvelar heart disease (total; 18,366)																	
	Valve	Cases	Operation					30-Day mortality				Hospital mortality		Redo			
			Mechanical	Bioprosthesis	Repair	Unknown	with CABG	Hospital		After discharge				Cases	30-Day mortality		Hospital mortality
								Replace	Repair	Replace	Repair	Replace	Repair		Hosipital	After discharge	
Isolated	A	8592	949	7465	125	53	2115	146 (1.7)	2 (1.6)	2 (0.02)	0	268 (3.2)	3 (2.4)	629	30 (4.8)	0	46 (7.3)
	M	4471	414	849	3177	31	607	50 (4.0)	17 (0.5)	1 (0.08)	1 (0.03)	97 (7.7)	36 (1.1)	622	15 (2.4)	0	39 (6.3)
	T	226	5	63	157	1	30	3 (4.4)	5 (3.2)	0	0	5 (7.4)	8 (5.1)	64	1 (1.6)	0	4 (6.3)
	P	11	0	11	0	0	1	0	2 (40)	0	0	0	0	10	0	0	0
A + M		1064					189	45 (4.2)		1 (0.09)		80 (7.5)		158	9 (5.7)	0	17 (10.8)
	A		202	827	31	4											
	M		140	381	538	5											
A + T		381					68	16 (4.2)		1 (0.3)		25 (6.6)		58	2 (3.4)	0	3 (5.2)
	A		48	331	2	0											
	T		0	0	371	10											
M + T		2818					292	48 (1.7)		1 (0.04)		92 (3.3)		358	9 (2.5)	1 (0.3)	19 (5.3)
	M		260	811	1734	13											
	T		8	24	2762	24											
A + M + T		756					104	25 (3.3)		0		50 (6.6)		104	4 (3.8)	1 (1.0)	9 (8.7)
	A		84	655	15	2											
	M		57	335	354	10											
	T		0	10	743	3											
Others		47					4	1 (2.1)		0		1 (2.1)		19	1 (5.3)	0	1 (5.3)
Total		18,366					3410	358 (1.9)		7 (0.04)		665 (3.6)		2022	71 (3.5)	2 (0.1)	138 (6.8)

	Cases	30-Day mortality	
TAVR	9774	99	(1.0)

(2) Ischemic heart disease (total, (A) + (B); 11,524)																					
(A) Isolated CABG (total; (a) + (b); 10,311)																					
(a-1) On-pump arrest CABG (total; 2263)																					
	Primary, elective				Primary, emergent				Redo, elective				Redo, emergent				Artery only	Artery + svg	svg only	Others	Unclear
	Cases	30 day mortality		Hospital mortality	Cases	30 day mortality		Hospital mortality	Cases	30 day mortality		Hospital mortality	Cases	30 day mortality		Hospital mortality					
		Hospital	After discharge			Hospital	After discharge			Hospital	After discharge			Hospital	After discharge						
1VD	36	1 (2.8)	0	1 (2.8)	12	2 (16.7)	0	2 (16.7)	1	0	0	0	0	0	0	0	20	14	15	0	0
2VD	257	4 (1.6)	0	7 (2.7)	39	3 (7.7)	0	6 (15.4)	3	0	0	0	1	1 (100.0)	0	1 (100.0)	34	235	30	1	0
3VD	847	8 (0.9)	0	14 (1.7)	80	2 (2.5)	0	6 (7.5)	3	1 (33.3)	0	1 (33.3)	1	0	0	0	30	869	28	4	0
LMT	792	15 (1.9)	0	21 (2.7)	152	11 (7.2)	0	17 (11.2)	3	0	0	0	2	0	0	0	83	822	38	6	0
No info	24	0	0	1 (4.2)	8	1 (12.5)	0	1 (12.5)	1	1 (100.0)	0	1 (100.0)	1	1 (100.0)	0	1 (100.0)	3	23	7	0	1
Total	1956	28 (1.4)	0	44 (2.2)	291	19 (6.5)	0	32 (11.0)	11	2 (18.2)	0	2 (18.2)	5	2 (40.0)	0	2 (40.0)	170	1963	118	11	1
Kawasaki	4	1 (25.0)	0	1 (25.0)	0	0	0	0	0	0	0	0	0	0	0	0	4	0	0	0	0
on dialysis	249	12 (4.8)	0	15 (6.0)	37	0	0	4 (10.8)	3	0	0	0	0	0	0	0	19	252	18	0	0
(), % mortality																					
*CABG* coronary artery bypass grafting, *1VD* one-vessel disease, *2VD* two-vessel disease, *3VD* three-vessel disease, *LMT* left main trunk, *SVG* saphenous vein graft																					
LMT includes LMT alone or LMT with other branch diseases																					

(a-2) On-pump beating CABG (total; 2034)																						
	Primary, elective					Primary, emergent				Redo, elective				Redo, emergent				Artery only	Artery + svg	svg only	Others	Unclear
	Cases	30 day mortality			Hospital mortality	Cases	30 day mortality		Hospital mortality	Cases	30 day mortality		Hospital mortality	Cases	30 day mortality		Hospital mortality					
		Hospital	After discharge				Hospital	After discharge			Hospital	After discharge			Hospital	After discharge						
1VD	30	1 (3.3)	0	0	1 (3.3)	9	1 (11.1)	0	2 (22.2)	2	0	0	0	1	0	0	0	18	14	10	0	0
2VD	211	3 (1.4)	0	0	5 (2.4)	36	4 (11.1)	0	6 (16.7)	2	0	0	0	0	0	0	0	56	172	18	3	0
3VD	679	13 (1.9)	0	0	20 (2.9)	133	14 (10.5)	0	21 (15.8)	9	0	0	1 (11.1)	1	0	0	0	93	697	26	6	0
LMT	664	14 (2.1)	1	0.150602	21 (3.2)	212	15 (7.1)	0	23 (10.8)	11	1 (9.1)	0	2 (18.2)	4	2 (50.0)	0	3 (75.0)	154	701	34	2	0
No info	21	0 (0.0)	0	0	0 (0.0)	6	3 (50.0)	0	3 (50.0)	0	0	0	0	3	0	0	1 (33.3)	9	11	8	2	0
Total	1605	31 (1.9)	1	0.062305	47 (2.9)	396	37 (9.3)	0 (0.0)	55 (13.9)	24	1 (4.2)	0	3 (12.5)	9	2 (22.2)	0	4 (44.4)	330	1595	96	13	0
Kawasaki	6	0	0		0	0	0	0	0	1	0	0	0	0	0	0	0	2	5	0	0	0
on dialysis	246	18 (7.3)	0		25 (10.2)	54	14 (25.9)	0 (0.0)	21 (38.9)	5	0 (0.0)	0	2 (40.0)	4	2 (50.0)	0	3 (75.0)	33	249	24	3	0
(), % mortality																						
*CABG* coronary artery bypass grafting, *1VD* one-vessel disease, *2VD* two-vessel disease, *3VD* three-vessel disease, *LMT* left main trunk, *SVG* saphenous vein graft																						
LMT includes LMT alone or LMT with other branch diseases																						

(b) Off-pump CABG (total; 6014)																						
(Including cases of planned off-pump CABG in which, during surgery, the change is made to an on-pump CABG or on-pump beating-heart procedure)																						
	Primary, elective				Primary, emergent					Redo, elective				Redo, emergent				Artery only	Artery + svg	svg only	Others	Unclear
	Cases	30 day mortality		Hospital mortality	Cases	30 day mortality			Hospital mortality	Cases	30 day mortality		Hospital mortality	Cases	30 day mortality		Hospital mortality					
		Hospital	After discharge			Hospital	After discharge				Hospital	After discharge			Hospital	After discharge						
1VD	340	0 (0.0)	0	1 (0.3)	28	3 (10.7)	0		3 (10.7)	5	0	0	0	1	0	0	0	256	76	41	0	1
2VD	836	2 (0.2)	0	9 (1.1)	63	1 (1.6)	0		3 (4.8)	6	0	0	0	0	0	0	0	301	579	21	3	1
3VD	2151	18 (0.8)	3 (0.1)	29 (1.3)	182	5 (2.7)	0		7 (3.8)	8	0	0	0 (0.0)	7	1 (14.3)	0	1 (14.3)	438	1857	32	21	0
LMT	1966	13 (0.7)	2 (0.1)	24 (1.2)	328	11 (3.4)	1	0.304878	18 (5.5)	15	0	0	0	3	0 (0.0)	0	0 (0.0)	615	1641	43	11	2
No info	50	0 (0.0)	0 (0.0)	1 (2.0)	19	0	0		3 (15.8)	4	0	0	0	2	2 (100.0)	0	2 (100.0)	26	41	8	0	0
Total	5343	33 (0.6)	5 (0.1)	64 (1.2)	620	20 (3.2)	1	0.16129	34 (5.5)	38	0	0	0 (0.0)	13	3 (23.1)	0	3 (23.1)	1636	4194	145	35	4
Kawasaki	11	0	0	0	1	0	0		0	0	0	0	0	0	0	0	0	5	7	0	0	0
on dialysis	584	4 (0.7)	1 (0.2)	12 (2.1)	56	2 (3.6)	0		4 (7.1)	6	0	0	0 (0.0)	2	0 (0.0)	0	0 (0.0)	139	482	25	2	0
(), % mortality																						
*CABG* coronary artery bypass grafting, *1VD* one-vessel disease, *2VD* two-vessel disease, *3VD* three-vessel disease, *LMT* left main trunk, *SVG* saphenous vein graft																						
LMT includes LMT alone or LMT with other branch diseases																						

(c) Cases of conversion, during surgery, from off-pump CABG to on-pump CABG or on- pump beating-heart CABG (these cases are also included in category (b))																
	Primary, elective				Primary, emergent				Redo, elective				Redo, emergent			
	Cases	30 day mortality		Hospital mortality	Cases	30 day mortality		Hospital mortality	Cases	30 day mortality		Hospital mortality	Cases	30 day mortality		Hospital mortality
		Hospital	After discharge			Hospital	After discharge			Hospital	After discharge			Hospital	After discharge	
Converted to arrest	24	2 (8.3)	0	3 (12.5)	5	2 (40.0)	0	2 (40.0)	0	0	0	0	0	0	0	0
Converted to beating	97	6 (6.2)	0	10 (10.3)	30	4 (13.3)	0	5 (16.7)	0	0	0	0	0	0	0	0
Total	121	8 (6.6)	0	13 (10.7)	35	6 (17.1)	0	7 (20.0)	0	0	0	0	0	0	0	0
On dialysis	36	4 (11.1)	0	7 (19.4)	5	1 (20.0)	0	2 (40.0)	0	0	0	0	0	0	0	0
(), % mortality																
*CABG* coronary artery bypass grafting																

(B) Operation for complications of MI (total; 1213)											
	Chronic				Acute				Concomitant operation		
	Cases	30-Day mortality		Hospital mortality	Cases	30-Day mortality		Hospital mortality			
		Hospital	After discharge			Hospital	After discharge		CABG	MVP	MVR
Infarctectomy or aneurysmectomy	119	8 (6.7)	0	10 (8.4)	26	4 (15.4)	0	7 (26.9)	80	31	2
VSP closure	85	9 (10.6)	0	13 (15.3)	250	67 (26.8)	0	87 (34.8)	92	0	8
Cardiac rupture	37	12 (32.4)	0	12 (32.4)	247	63 (25.5)	0	80 (32.4)	46	3	9
Mitral regurgitation											
(1) Papillary muscle rupture	9	0	0	0	51	10 (19.6)	0	16 (31.4)	26	4	56
(2) Ischemic	204	12 (5.9)	0	24 (11.8)	39	8 (20.5)	0	12 (30.8)	191	138	105
Others	69	1 (1.4)	0	4 (5.8)	77	25 (32.5)	0	27 (35.1)	51	8	5
Total	523	42 (8.0)	0	63 (12.0)	690	177 (25.7)	0	229 (33.2)	486	184	185
(), % mortality											
*MI* myocardial infarction; CABG, coronary artery bypass grafting; MVP, mitral valve repair; MVR, mitral valve replacement; VSP, ventricular septal perforation											
Acute, within 2 weeks from the onset of myocardial infarction											

(3) Operation for arrhythmia (total; 6831)											
	Cases	30-Day mortality		Hospital mortality	Concomitant operation						
					Isolated	Congenital	Valve	IHD	Others	Multiple combination	
		Hospital	After discharge							2 categories	3 categories
Maze	3680	55 (1.5)	1 (0.03)	104 (2.8)	204	158	3126	631	344	728	45
For WPW	0	0	0	0	0	0	0	0	0	0	0
For ventricular tachyarrhythmia	23	1 (4.3)	0	1 (4.3)	5	0	10	10	1	0	0
Others	3128	60 (1.9)	1 (0.03)	117 (3.7)	68	139	2625	575	378	626	42
Total	6831	116 (1.7)	2 (0.03)	222 (3.2)	277	297	5761	1216	723	1354	87
(), % mortality											
*WPW* Wolff–Parkinson–White syndrome, *IHD* ischemic heart disease											
Except for 170 isolated cases, all remaining 5164 cases are doubly allocated, one for this subgroup and the other for the subgroup corresponding to the concomitant operations											

(4) Operation for constrictive pericarditis (total; 210)								
	CPB (+)				CPB (−)			
	Cases	30-Day mortality		Hospital mortality	Cases	30-Day mortality		Hospital mortality
		Hospital	After discharge			Hospital	After discharge	
Total	119	7 (5.9)	0	18 (15.1)	91	3 (3.3)	1 (1.1)	7 (7.7)
(), % mortality								
*CPB* cardiopulmonary bypass								

(5) Cardiac tumor (total; 628)								
	Cases	30-Day mortality		Hospital mortality	Concomitant operation			
		Hospital	After discharge		AVR	MVR	CABG	Others
Benign tumor	537	4 (0.7)	0	4 (0.7)	30	23	60	116
(Cardiac myxoma)	353	0	0	0	9	7	33	67
Malignant tumor	91	6 (6.6)	1 (1.1)	9 (9.9)	3	3	1	9
(Primary)	48	3 (6.3)	0	4 (8.3)	1	3	1	5
(), % mortality								
*AVR* aortic valve replacement, *MVR* mitral valve replacement, *CABG* coronary artery bypass grafting								

(6) HOCM and DCM (total; 264)								
	Cases	30-Day mortality		Hospital mortality	Concomitant operation			
		Hospital	After discharge		AVR	MVR	MVP	CABG
Myectomy	118	3 (2.5)	0	5 (4.2)	41	17	24	10
Myotomy	5	0	0	1 (20.0)	1	1	1	0
No-resection	133	7 (5.3)	0	10 (7.5)	27	60	73	14
Volume reduction surgery of the left ventricle	8	0	0	0	0	1	2	2
Total	264	10 (3.8)	0	16 (6.1)	69	79	100	26
(), % mortality								
*HOCM* hypertrophic obstructive cardiomyopathy, *DCM* dilated cardiomyopathy, *AVR* aortic valve replacement, *MVR* mitral valve replacement, *MVP* mitral valve repair, *CABG* coronary artery bypass grafting								

(7) Other open-heart operation (total; 1240)				
	Cases	30-Day mortality		Hospital mortality
		Hospital	After discharge	
Open-heart operation	511	56 (11.0)	0	83 (16.2)
Non-open-heart operation	729	81 (11.1)	0	114 (15.6)
Total	1240	137 (11.0)	0	197 (15.9)

(), % mortality

**Table 3:** Thoracic aortic aneurysm (total; 22,540) (1) Dissection (total; 10,855)

Stanford type	Acute								Chronic									Concomitant operation					
	A				B				A					B									
Replaced site	Cases	30-Day mortality		Hospital mortality	Cases	30-Day mortality		Hospital mortality	Cases	30-Day mortality			Hospital mortality	Cases	30-Day mortality		Hospital mortality	AVP	AVR	MVP	MVR	CABG	Others
		Hospital	After discharge			Hospital	After discharge			Hospital		After discharge			Hospital	After discharge							
Ascending Ao	2071	146 (7.0)	1 (0.05)	189 (9.1)	1	0	0	0	187	6 (3.2)		0	8 (4.3)	1	0	0	0	61	137	22	8	110	32
Aortic Root	191	35 (18.3)	0	36 (18.8)	0	0	0	0	80	4 (5.0)		0	5 (6.3)	3	0	0	0	32	194	6	2	65	7
Arch	1954	135 (6.9)	1 (0.05)	174 (8.9)	31	0	0	0	355	9 (2.5)		0	13 (3.7)	172	5 (2.9)	0	6 (3.5)	54	113	10	5	118	25
Aortic root + asc. Ao. + Arch	167	23 (13.8)	0	26 (15.6)	0	0	0	0	47	1 (2.1)		0	3 (6.4)	6	0	0	0	23	143	2	0	35	2
Descending Ao	35	4 (11.4)	0	4 (11.4)	28	2 (7.1)	0	2 (7.1)	56	1 (1.8)		0	1 (1.8)	220	9 (4.1)	0	10 (4.5)	2	4	0	0	4	0
Thoracoabdominal	1	1 (100.0)	0	1 (100.0)	11	1 (9.1)	0	1 (9.1)	46	5 (10.9)		0	5 (10.9)	182	11 (6.0)	1 (0.5)	13 (7.1)	0	0	0	0	1	0
Simple TEVAR	101	9 (8.9)	0	11 (10.9)	442	30 (6.8)	0	34 (7.7)	264	2 (0.8)		0	3 (1.1)	1171	7 (0.6)	0	8 (0.7)	1	2	0	0	2	2
Open SG with BR	1213	101 (8.3)	2 (0.16)	133 (11.0)	62	3 (4.8)	0	3 (4.8)	207	8 (3.9)		0	11 (5.3)	237	4 (1.7)	0	7 (3.0)	61	115	10	2	104	16
Open SG without BR	435	32 (7.4)	0	45 (10.3)	28	2 (7.1)	0	3 (10.7)	52	2 (3.8)		0	4 (7.7)	82	3 (3.7)	0	3 (3.7)	20	45	1	0	30	2
Arch TEVAR with BR	14	1 (7.1)	0	1 (7.1)	123	6 (4.9)	0	10 (8.1)	73	2 (2.7)		0	2 (2.7)	364	7 (1.9)	0	8 (2.2)	1	0	0	0	0	0
Thoracoabdominal TEVAR with BR	0	0	0	0	11	0	0	0	6	0		0	1 (16.7)	33	2 (6.1)	0	4 (12.1)	0	0	0	0	0	0
Other	18	6 (33.3)	0	6 (33.3)	13	3 (23.1)	0	3 (23.1)	10	0		0	0	51	2 (3.9)	0	2 (3.9)	0	2	0	0	1	1
Total	6200	353 (5.7)	4 (0.06)	626 (10.1)	750	47 (6.3)	0	56 (7.5)	1383	40	(2.9)	0	56 (4.0)	2522	50 (2.0)	1 (0.0)	61 (2.4)	255	755	51	17	470	87
(), % mortality																							
*Ao* aorta, *AVP* aortic valve repair, *AVR* aortic valve replacement, *MVP* mitral valve repair, *MVR* mitral valve replacement, *CABG* coronary artery bypass grafting, *TEVAR* thoracic endovascular aortic (aneurysm) repair																							
Acute, within 2 weeks from the onset																							

(2) Non-dissection (total; 11,685)														
Replaced site	Unruptured				Ruptured				Concomitant operation					
	Cases	30-Day mortality		Hospital mortality	Cases	30-Day mortality		Hospital mortality	AVP	AVR	MVP	MVR	CABG	Others
		Hospital	After discharge			Hospital	After discharge							
Ascending Ao	1423	33 (2.3)	0	51 (3.6)	56	7 (12.5)	0	11 (19.6)	43	1055	65	68	181	115
Aortic Root	1056	22 (2.1)	0	35 (3.3)	59	10 (16.9)	0	11 (18.6)	243	783	73	27	144	77
Arch	2035	38 (1.9)	0	67 (3.3)	113	10 (8.8)	0	14 (12.4)	33	569	37	23	304	76
Aortic root + asc. Ao. + Arch	306	10 (3.3)	0	14 (4.6)	10	0	0	2 (20.0)	53	225	12	0	39	10
Descending Ao	305	5 (1.6)	2 (0.66)	14 (4.6)	32	5 (15.6)	0	5 (15.6)	1	8	0	0	16	3
Thoracoabdominal	377	14 (3.7)	0	27 (7.2)	45	5 (11.1)	0	8 (17.8)	0	0	0	0	0	0
Simple TEVAR	2457	32 (1.3)	5 (0.20)	55 (2.2)	373	56 (15.0)	1 (0.27)	74 (19.8)	0	2	0	0	1	5
Open SG with BR	1115	40 (3.6)	0	68 (6.1)	66	7 (10.6)	0	13 (19.7)	7	121	13	2	166	12
Open SG without BR	398	8 (2.0)	0	24 (6.0)	33	3 (9.1)	0	7 (21.2)	6	67	7	2	55	8
Arch TEVAR with BR	1080	21 (1.9)	3	33 (3.1)	58	8 (13.8)	0	9 (15.5)	0	0	1	0	3	2
Thoracoabdominal TEVAR with BR	107	9 (8.4)	1 (0.93)	11 (10.3)	15	1 (6.7)	0	2 (13.3)	0	0	0	0	0	0
Other	137	2 (1.5)	0	6 (4.4)	29	7 (24.1)	0	8 (27.6)	0	14	2	0	8	4
Total	10,796	234 (2.2)	11 (0.10)	405 (3.8)	889	119 (13.4)	1 (0.11)	164 (18.4)	386	2844	210	122	917	312

(), % mortality

*Ao* aorta, *AVP* aortic valve repair, *AVR* aortic valve replacement, *MVP* mitral valve repair, *MVR* mitral valve replacement, *CABG* coronary artery bypass grafting, *TEVAR* thoracic endovascular aortic (aneurysm) repair

**Table 4:** Pulmonary thromboembolism (total; 190)

	Cases	30-Day mortality		Hospital mortality
		Hospital	After discharge	
Acute	131	25 (19.1)		30 (22.9)
Chronic	59	0		1 (1.7)
Total	190	25 (13.2)	0	31 (16.3)

(), % mortality

**Table 5:** Implantation of VAD (total; 187)

	Cases	30-Day mortality		Hospital mortality
		Hospital	After discharge	
Implantation of VAD	187	2 (1.1)	0	14 (7.5)

(), % mortality

*VAD* ventricular assist devise

**Table 6:** Heart transplantation (total; 54)

	Cases	Age	
		< 18 years	18 years ≤
Heart transplantation	54	5	49
Heart and lung transplantation	0	0	0
Total	54	5	49

(), % mortality

Among the 8595 procedures for congenital heart disease conducted in 2020, 6543 were open-heart surgeries, with an overall hospital mortality rate of 2.0%. The number of surgeries for neonates and infants in 2020 did not significantly differ compared to that in 2010; however, hospital mortality improved from 11.5 to 8.2% for neonates and from 3.0 to 2.1% for infants. In 2020, atrial septal defect was the most common disease (1188 cases) as previously reported, with patients aged ≥ 18 years accounting for 63% of atrial septal defect surgery. Ventricular septal defect (perimembranous/muscular), which had been the most common disease in 2015 and 2016, was the second most common disease (1043 cases).

Hospital mortality for complex congenital heart disease within the past 10 years was as follows (2010 [4], 2015 [5], and 2020): complete atrioventricular septal defect (4.2%, 5.4%, and 2.2%); tetralogy of Fallot (0.8%, 2.1%, and 0.8%); transposition of the great arteries with the intact septum (4.1%, 7.1%, and 0%), ventricular septal defect (7.4%, 7.1%, and 0%), and single ventricle (7.5%, 3.9%, and 5.9%); and hypoplastic left heart syndrome (13.1%, 8.0%, and 9.3%). Currently, right heart bypass surgery has been commonly performed (335 bidirectional Glenn procedures, excluding 35 Damus–Kaye–Stansel procedures, and 378 Fontan type procedures, including total cavopulmonary connection) with acceptable hospital mortality rates (1.2% and 0.8%). The Norwood type I procedure was performed in 117 cases, with a relatively low hospital mortality rate (16.2%).

Valvular heart disease procedures, excluding transcatheter procedures, were performed less than that in the previous year. Isolated aortic valve replacement/repair with/without coronary artery bypass grafting (CABG) (*n* = 8592) was 16.3%% fewer than that in the previous year (*n* = 10,268) and 0.7% fewer than that 5 years ago (*n* = 8651), as opposed to the rapid increase of transcatheter aortic valve replacement (*n* = 9774 in 2020). Isolated mitral valve replacement/repairs with/without CABG (*n* = 4471) was 14.7% fewer than that in the previous year (*n* = 5239) and 1.2% fewer than that 5 years ago (*n* = 4524). Aortic and mitral valve replacement with bioprosthesis were performed in 9278 and 2376 cases, respectively. The rate at which bioprosthesis was used had dramatically increased from 30% in the early 2000s [6, 7] to 87.9% and 72.6% in 2020 for aortic and mitral positions, respectively. Additionally, CABG was performed concurrently in 18.6% of all valvular procedures (17.8% in 2010 [4] and 19.8% in 2015 [5]). Valve repair was common in mitral and tricuspid valve positions (5803 and 4033 cases, respectively) but less common in aortic valve positions (173 patients, only 1.6% of all aortic valve procedures). Mitral valve repair accounted for 63.7% of all mitral valve procedures. Hospital mortality rates for single valve replacement for aortic and mitral positions were 3.2% and 7.7%, respectively, but only 1.1% for mitral valve repair. Moreover, hospital mortality rates for redo valve surgery for the aortic and mitral positions were 7.3% and 6.3%, respectively. Finally, overall hospital mortality rates did not significantly improve over the past 10 years (3.4% in 2010 [4], 4.0% in 2015 [5], and 3.6% in 2020).

Isolated CABG had been performed in 10,311 cases, accounting for only 66.4% of the procedures performed 10 years ago (*n* = 15,521) [4]. Of the aforementioned cases, 6014 (58.3%) underwent off-pump CABG, with a success rate of 98.0%. The percentage of planned off-pump CABG in 2020 was similar to that in 2019. Hospital mortality associated with primary elective CABG procedures among 8904 cases accounted for 1.7%, which is slightly higher than that in 2010 (1.1%) [4]. Hospital mortality for primary emergency CABG among 1307 cases remained high (9.2%). The percentage of conversion from off-pump to on-pump CABG or on-pump beating-heart CABG was 2.3% among the primary elective CABG cases, with a hospital mortality rate of 10.7%. Patients with end-stage renal failure on dialysis had higher hospital mortality rates than overall mortality, regardless of surgical procedure (on-pump arrest, on-pump beating, and off-pump). This study excluded concomitant CABGs alongside other major procedures under the ischemic heart disease category but rather under other categories, such as valvular heart disease and thoracic aortic aneurysm. Accordingly, the overall number of CABGs in 2020, including concomitant CABG with other major procedures, was 15,681.

Arrhythmia management was primarily performed as concomitant procedures in 6831 cases, with a hospital mortality rate of 3.2%. Pacemaker and implantable cardioverter-defibrillator implantation were not included in this category.

In 2020, 22,540 procedures for thoracic and thoracoabdominal aortae diseases were performed, among which aortic dissection and non-dissection accounted for 10,855 and 11,685, respectively. The number of surgeries for aortic dissection this year was 0.1% higher than that in the preceding year (*n* = 10,847). Hospital mortality rates for the 6200 Stanford type A acute aortic dissections remained high (10.1%). The number of procedures for non-dissected aneurysms decreased by 1.5%, with a hospital mortality rate of 4.9% for all aneurysms and 3.8% and 18.4% for unruptured and ruptured aneurysms, respectively. Thoracic endovascular aortic repair (TEVAR) has been performed for aortic diseases at an increasing rate. Stent graft placement was performed in 4918 patients with aortic dissection, including 2602 TEVARs and 2316 open stent graftings. Moreover, 1568 and 319 cases underwent TEVAR and open stent grafting for type B chronic aortic dissection, accounting for 62.2% and 12.6% of the total number of cases, respectively. Hospital mortality rates associated with simple TEVAR for type B aortic dissection were 7.7% and 0.7% for acute and chronic cases, respectively. Stent graft placement was performed in 5702 patients with non-dissected aortic aneurysms, among which 4090 were TEVARs (an 0.4% increase compared to that in 2019, *n* = 4072) and 1612 were open stent graftings (a 7.5% increase compared to that in 2019, *n* = 1499). Hospital mortality rates were 2.7% and 19.1% for TEVARs and 6.1% and 20.2% for open stenting in unruptured and ruptured aneurysms, respectively.

### (B) General thoracic surgery

**Fig. 2:**
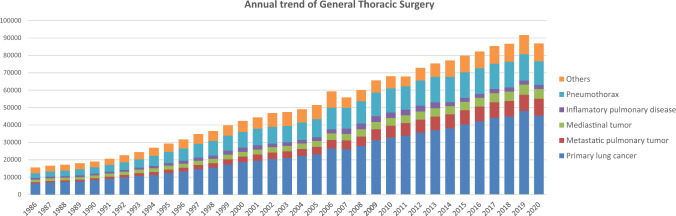
Annual trend of general thoracic surgery

The 2020 survey of general thoracic surgeries comprised 708 surgical units, with bulk data submitted via a web-based collection system established by the NCD [3]. General thoracic surgery departments reported 86,813 procedures in 2020 (Table 7), which is 2.1 times more than that in 2000 and approximately 7038 more procedures than that in 2015 (Fig. 2). However it decreased by 5.3% compared to that of 2019 (91,626), mostly because of COVID-19 pandemic, despite the steadily increase up to 2019.

**Table 7:** Total cases of general thoracic surgery during 2020

	Cases	%
Benign pulmonary tumor	2232	2.6
Primary lung cancer	45,436	52.3
Other primary malignant pulmonary tumor	336	0.4
Metastatic pulmonary tumor	9654	11.1
Tracheal tumor	98	0.1
Pleural tumor including mesothelioma	584	0.7
Chest wall tumor	652	0.8
Mediastinal tumor	5573	6.4
Thymectomy for MG without thymoma	130	0.1
Inflammatory pulmonary disease	2397	2.8
Empyema	3138	3.6
Bullous disease excluding pneumothorax	317	0.4
Pneumothorax	13,514	15.6
Chest wall deformity	180	0.2
Diaphragmatic hernia including traumatic	41	0.0
Chest trauma excluding diaphragmatic hernia	458	0.5
Lung transplantation	75	0.1
Others	1998	2.3
Total	86,813	100.0

In 2020, 45,436 procedures for primary lung cancer had been performed which decreased by 5.4% compared to that of 2019 (48,052) similarly to the total number of surgeries in general thoracic surgery. The number of procedures in 2020 was 2.4 times higher than that in 2000, with lung cancer procedures accounting for 52% of all general thoracic surgeries.

Information about the number of video-assisted thoracoscopic surgery (VATS), which is defined as surgical procedures using a skin incision less than 8 cm including a mini-thoracotomy (hybrid) approach, have been available since the 2015 annual report. Tables 8, 9, 11, 14, 15, 16, 18, 19, 20, 21, 22, and 24, 25, 26 present the number of VATS procedures for benign pulmonary tumors, primary lung cancer, metastatic pulmonary tumor, chest wall tumor, mediastinal tumor, thymectomy for myasthenia gravis, inflammatory pulmonary disease, empyema, descending necrotizing mediastinitis, bullous diseases, pneumothorax, diaphragmatic hernia, chest trauma and other respiratory surgeries in 2020, respectively.

**Table 8:** Benign pulmonary tumor

	Cases	30-Day mortality		Hospital mortality	by VATS
		Hospital	After discharge		
1. Benign pulmonary tumor					
Hamartoma	443	0	0	0	429
Sclerosing hemangioma	95	0	1 (1.1)	0	88
Papilloma	20	0	0	0	19
Mucous gland adenoma bronchial	12	0	0	0	11
Fibroma	133	0	0	1 (0.8)	123
Lipoma	9	0	0	0	7
Neurogenic tumor	14	0	0	0	14
Clear cell tumor	3	0	0	0	3
Leiomyoma	22	0	0	0	21
Chondroma	8	0	0	0	8
Inflammatory myofibroblastic tumor	0	0	0	0	0
Pseudolymphoma	22	0	0	0	20
Histiocytosis	16	0	0	0	14
Teratoma	4	0	0	0	4
Others	1431	0	0	1 (0.1)	1318
Total	2232	0	1 (0.04)	2 (0.09)	2079

(), Mortality %

**Table 9:** Primary malignant pulmonary tumor

	Cases	30-Day mortality		Hospital mortality	VATS	Robotic surgery
		Hospital	After discharge			
2. Primary malignant pulmonary tumor	45,772	122 (0.3)	45 (0.1)	235 (0.5)	33,992	3078
Lung cancer	45,436	122 (0.3)	45 (0.1)	235 (0.5)	33,992	3078
Adenocarcinoma	31,632	55 (0.2)	21 (0.07)	97 (0.3)		
Squamous cell carcinoma	8217	44 (0.5)	16 (0.2)	98 (1.2)		
Large cell carcinoma	288	2 (0.7)	0	2 (0.7)		
LCNEC	573	7 (1.2)	3 (0.5)	10 (1.7)		
Small cell carcinoma	888	4 (0.5)	2 (0.2)	9 (1.0)		
Adenosquamous carcinoma	565	1 (0.2)	0	3 (0.5)		
Carcinoma with pleomorphic, sarcomatoid or sarcomatous elements	553	2 (0.4)	0	6 (1.1)		
Carcinoid	249	0	0	0		
Carcinomas of salivary-gland type	18	0	0	0		
Unclassified	39	0	1 (2.6)	0		
Multiple lung cancer	2061	4 (0.2)	2 (0.1)	7 (0.3)		
Others	306	3 (1.0)	0	3 (1.0)		
Wedge resection	8511	10 (0.1)	8 (0.1)	24 (0.3)	7815	12
Segmental excision	5794	10 (0.2)	2 (0.03)	15 (0.3)	4784	253
(*Sleeve segmental excision*)	10	0	0	0	4	0
Lobectomy	30,604	94 (0.3)	35 (0.11)	182 (0.6)	21,179	2810
(*Sleeve lobectomy*)	396	2 (0.5)	0	9 (2.3)	51	1
Pneumonectomy	251	5 (2.0)	0	9 (3.6)	35	2
(*Sleeve pneumonectomy*)	8	1 (12.5)	0	1 (12.5)	0	0
Other bronchoplasty	32	1 (3.1)	0	1 (3.1)	4	0
Pleuropneumonectomy	1	0	0	0	0	0
Others	198	2 (1.0)	0	4 (2.0)	135	1
Multiple incision for Multiple lung cancer	45	0	0	0	40	0
Sarcoma	53	0	0	0		
AAH	11	0	0	0		
Others	272	0	0	0		

(), Mortality %

A total of 2232 procedures for benign pulmonary tumors had been conducted in 2020 (Table 8). Hamartomas were the most frequent benign pulmonary tumors diagnosed, with 2079 patients (93%) undergoing VATS.

Tables 9 and 10 show additional information on primary malignant pulmonary tumors. Accordingly, the most frequently diagnosed lung cancer subtype was adenocarcinoma (70% of all lung cancers), followed by squamous cell carcinoma (18%). Sublobar resection was performed in 14,305 lung cancer cases (31% of all cases) and lobectomy in 30,604 cases (67% of all cases). Sleeve lobectomy was performed in 396 cases (0.9% of all cases), while pneumonectomy was required in 251 cases (0.6% of all cases). VATS lobectomy was performed in 21,179 cases of lung cancer (69% of all lobectomy cases). RATS lobectomy was performed in 2810 cases of lung cancer (9% of all lobectomy cases). Patients aged ≥ 80 years who underwent lung cancer surgery accounted for 6521 (14%). Among those who died within 30 days postoperatively, 122 and 45 died before and after hospital discharge, respectively. Overall, 167 patients died within 30 days postoperatively (30-day mortality rate, 0.4%), while 122 died before discharge (hospital mortality rate, 0.3%). Moreover, 30-day mortality rates according to the procedure were 0.2%, 0.4%, and 2% for segmentectomy, lobectomy, and pneumonectomy, respectively. Interstitial pneumonia had been the leading cause of death after lung cancer surgery, followed by pneumonia, respiratory failure, and cardiovascular events.

**Table 10:** Details of lung cancer operations

TNM	
c-Stage	Cases
IA1	8499
IA2	13,478
IA3	7783
IB	4886
IIA	1487
IIB	3746
IIIA	2448
IIIB	444
IIIC	19
IVA	367
IVB	105
NA	2129
Total	45,391

Sex	Cases
Male	27,831
Female	17,560
NA	0
Total	45,391

Cause of death	Cases
Cardiovascular	38
Pneumonia	96
Pyothorax	4
Bronchopleural fistula	11
Respiratory failure	32
Pulmonary embolism	4
Interstitial pneumonia	101
Brain infarction or bleeding	21
Others	140
Unknown	25
Total	472

p-Stage	Cases
0 (pCR)	3124
IA1	9234
IA2	10,515
IA3	4957
IB	6300
IIA	1177
IIB	4475
IIIA	3594
IIIB	780
IIIC	11
IVA	866
IVB	99
NA	257
Total	45,389

Age (y)	Cases
< 20	19
20–29	39
30–39	232
40–49	1142
50–59	3595
60–69	11,483
70–79	22,360
80–89	6422
≥ 90	99
NA	0
Total	45,391

The procedures for metastatic pulmonary tumors performed in 2020 increased 3.4% to 9654 cases compared to that in 2019 (9329), which showed contrastive trend to primary lung cancer (Table 11). Among such procedures, the most frequent primary tumor was colorectal cancer (48% of all cases).

**Table 11:** Metastatic pulmonary tumor

	Cases	30-Day mortality		Hospital mortality	VATS
		Hospital	After discharge		
3. Metastatic pulmonary tumor	9654	11 (0.1)	7 (0.07)	21 (0.2)	8784
Colorectal	4633	3 (0.06)	1 (0.02)	4 (0.1)	4232
Hepatobiliary/pancreatic	528	2 (0.4)	1 (0.2)	4 (0.8)	489
Uterine	512	0	1 (0.2)	0	476
Mammary	549	0	2 (0.4)	0	515
Ovarian	80	0	0	0	72
Testicular	59	0	0	0	51
Renal	768	0	0	0	712
Skeletal	115	0	0	0	100
Soft tissue	257	0	0	1 (0.4)	220
Otorhinolaryngological	517	0	2 (0.4)	1 (0.2)	480
Pulmonary	537	2 (0.4)	0	3 (0.6)	431
Others	1099	4 (0.4)	0	8 (0.7)	1006

(), Mortality %

A total of 98 procedures for tracheal tumors, including 49, 30, and 19 cases of primary malignant, metastatic, and benign tracheal tumors, respectively, were performed in 2020. Further, 17 patients underwent sleeve resection and reconstruction (Table 12).

**Table 12:** Tracheal tumor

	Cases	30-Day mortality		Hospital mortality
		Hospital	After discharge	
4. Tracheal tumor	98	1 (1.0)	2 (2.0)	1 (1.0)
A. Primary malignant tumor				
Histological classification				
Squamous cell carcinoma	17	0	1 (5.9)	0
Adenoid cystic carcinoma	17	0	0	0
Mucoepidermoid carcinoma	2	0	0	0
Others	13	0	0	0
Total	49	0	1 (2.0)	0
B. Metastatic/invasive malignant tumor, e.g. invasion of thyroid cancer				
	30	0	1 (3.3)	1 (3.3)
C. Benign tracheal tumor				
Histological classification				
Papilloma	3	0	0	0
Adenoma	2	0	0	0
Neurofibroma	0	0	0	0
Chondroma	0	0	0	0
Leiomyoma	1	0	0	0
Others	13	0	0	0
Histology unknown	0	0	0	0
Total	19	0	0	0
Operation				
Sleeve resection with reconstruction	17	0	0	0
Wedge with simple closure	2	0	0	0
Wedge with patch closure	0	0	0	0
Total laryngectomy with tracheostomy	0	0	0	0
Others	2	0	0	0
Unknown	0	0	0	0
Total	21	0	0	0

(), Mortality %

Overall, 584 pleural tumors had been diagnosed in 2020 (Table 13), with diffuse malignant pleural mesothelioma as the most frequent histologic diagnosis. Total pleurectomy was performed in 105 cases and extrapleural pneumonectomy in 33 cases. The 30-day mortality rate was 2% and 3% after total pleurectomy and extrapleural pneumonectomy, respectively.

**Table 13:** Tumor of pleural origin

5. Tumor of pleural origin				
Histological classification	Cases	30-Day mortality		Hospital mortality
		Hospital	After discharge	
Solitary fibrous tumor	106	0	0	0
Diffuse malignant pleural mesothelioma	213	4 (1.9)	0	7 (3.3)
Localized malignant pleural mesothelioma	32	0	0	1 (3.1)
Others	233	0	2 (0.9)	4 (1.7)
Total	584	4 (0.7)	2 (0.3)	12 (2.1)

Operative procedure	Cases	30-Day mortality		Hospital mortality
		Hospital	After discharge	
Extrapleural pneumonectomy	33	1 (3.0)	0	2 (6.1)
Total pleurectomy	105	2 (1.9)	0	3 (2.9)
Others	75	1 (1.3)	1 (1.3)	2 (2.7)
Total	213	4 (1.9)	1 (0.5)	7 (3.3)

(), Mortality %

Overall, 652 chest wall tumor resections had been performed in 2020, including 103, 209, and 340 cases of primary malignant, metastatic, and benign tumors, respectively (Table 14).

**Table 14:** Chest wall tumor

	Cases	30-Day mortality		Hospital mortality	VATS
		Hospital	After discharge		
6. Chest wall tumor					
Primary malignant tumor	103	0	0	0	42
Metastatic malignant tumor	209	0	1 (0.5)	0	66
Benign tumor	340	0	0	0	251
Total	652	0	1 (0.2)	0	359

(), Mortality %

In 2020, 5573 mediastinal tumors were resected, which decreased by 5% compared to that in 2019 (5881) (Table 15), which showed similar trend as primary lung cancer. Thymic epithelial tumors, including 2226 thymomas, 341 thymic carcinomas, and 48 thymic carcinoids, were the most frequently diagnosed mediastinal tumor subtype in 2020.

**Table 15:** Mediastinal tumor

	Cases	30-Day mortality		Hospital mortality	By VATS	Robotic surgery
		Hospital	After discharge			
7. Mediastinal tumor	5573	5 (0.09)	0	9 (0.2)	4224	938
Thymoma*	2226	0	0	3 (0.1)	1511	366
Thymic cancer	341	0	0	0	186	37
Thymus carcinoid	48	0	0	0	27	6
Germ cell tumor	86	1 (1.2)	0	1 (1.2)	54	13
* Benign*	68	1 (1.5)	0	1 (1.5)	48	11
* Malignant*	18	0	0	0	6	2
Neurogenic tumor	393	0	0	0	373	77
Congenital cyst	1239	0	0	0	1164	270
Goiter	73	0	0	0	27	5
Lymphatic tumor	168	1 (0.6)	0	1 (0.6)	124	16
Excision of pleural recurrence of thymoma	30	0	0	0	20	1
Thymolipoma	19	0	0	0	8	1
Others	950	3 (0.3)	0	4 (0.4)	730	146

(), Mortality %

A total of 484 patients underwent thymectomy for myasthenia gravis (Table 16), among which 354 procedures were associated with thymoma in 2020.

**Table 16:** Thymectomy for myasthenia gravis

	Cases	30-Day mortality		Hospital mortality	By VATS	Robotic surgery
		Hospital	After discharge			
8. Thymectomy for myasthenia gravis	484	0	0	2 (0.4)	319	19
With thymoma	354	0	0	2 (0.6)	209	2

(), Mortality %

Overall, 22,043 patients underwent procedures for non-neoplastic disease. Accordingly, 2397 patients underwent lung resection for inflammatory lung diseases (Tables 17, 18), among which 492 and 311 patients were associated with mycobacterial and fungal infections, respectively. Procedures for inflammatory pseudotumor were performed in 1011 cases (42%).

**Table 17:** Operations for non-neoplastic diseases: A + B + C + D + E + F + G + H + I

	Cases	30-Day mortality		Hospital mortality
		Hospital	After discharge	
9. Operations for non-neoplastic diseases	22,043	250 (1.1)	48 (0.2)	502 (2.3)

**Table 18:** A. Inflammatory pulmonary disease

	Cases	30-Day mortality		Hospital mortality	VATS
		Hospital	After discharge		
A. Inflammatory pulmonary disease	2397	9 (0.4)	3 (0.1)	26 (1.1)	2047
Tuberculous infection	43	0	0	0	33
Mycobacterial infection	492	3 (0.6)	0	3 (0.6)	443
Fungal infection	311	1 (0.3)	0	11 (3.5)	203
Bronchiectasis	45	1 (2.2)	0	2 (4.4)	30
Tuberculous nodule	51	0	0	0	42
Inflammatory pseudotumor	1011	0	0	2 (0.2)	936
Interpulmonary lymph node	57	0	0	0	52
Others	387	4 (1.0)	3 (0.8)	8 (2.1)	308

(), Mortality %

A total of 3138 procedures were performed for empyema (Table 19), among which 2456 (78%) were acute and 682 (22%) were chronic. Further, bronchopleural fistulas developed in 465 and 346 patients with acute and chronic empyema, respectively. The hospital mortality rate was 16% among patients with acute empyema with fistula.

**Table 19:** B. Empyema

	Cases	30-Day mortality		Hospital mortality	By VATS
		Hospital	After discharge		
Acute empyema	2456	67 (2.7)	7 (0.3)	133 (5.4)	2002
With fistula	465	32 (6.9)	3 (0.6)	72 (15.5)	241
Without fistula	1952	34 (1.7)	4 (0.2)	59 (3.0)	1727
Unknown	39	1 (2.6)	0	2 (5.1)	34
Chronic empyema	682	22 (3.2)	3 (0.4)	63 (9.2)	321
With fistula	346	15 (4.3)	1 (0.3)	37 (10.7)	116
Without fistula	303	7 (2.3)	2 (0.7)	23 (7.6)	180
Unknown	33	0	0	3 (9.1)	25
Total	3138	89 (2.8)	10 (0.3)	196 (6.2)	2323

(), Mortality %

Further, 99 operations were performed for descending necrotizing mediastinitis (Table 20), with a hospital mortality rate of 6%.

**Table 20:** C. Descending necrotizing mediastinitis

	Cases	30-Day mortality		Hospital mortality	VATS
		Hospital	After discharge		
C. Descending necrotizing mediastinitis	99	4 (4.0)	0	6 (6.1)	76

(), Mortality %

A total of 317 procedures were conducted for bullous diseases (Table 21), while only 14 patients underwent lung volume reduction surgery.

**Table 21:** D. Bullous diseases

	Cases	30-Day mortality		Hospital mortality	VATS
		Hospital	After discharge		
D. Bullous diseases	317	1 (0.3)	0	2 (0.6)	285
Emphysematous bulla	235	1 (0.4)	0	2 (0.9)	219
Bronchogenic cyst	10	0	0	0	8
Emphysema with LVRS	14	0	0	0	12
Others	58	0	0	0	46

(), Mortality %

*LVRS* lung volume reduction surgery

A total of 13,514 procedures were performed for pneumothorax (Table 22). Among the 9592 procedures for spontaneous pneumothorax, 2523 (26%) were bullectomies alone, while 6428 (67%) required additional procedures, such as coverage with artificial material, as well as parietal pleurectomy. A total of 3922 procedures for secondary pneumothorax were performed, with chronic obstructive pulmonary disease (COPD) being the most prevalent associated disease (2775 cases, 71%). The hospital mortality rate for secondary pneumothorax associated with COPD was 3%.

**Table 22:** E. Pneumothorax

Cases	30-Day mortality		Hospital mortality	VATS
	Hospital	After discharge		
13,514	67 (0.5)	27 (0.2)	146 (1.1)	13,115

Spontaneous pneumothorax					
Operative procedure	Cases	30-Day mortality		Hospital mortality	VATS
		Hospital	After discharge		
Bullectomy	2523	1 (0.0)	3 (0.1)	6 (0.2)	2770
Bullectomy with additional procedure	6428	4 (0.1)	4 (0.06)	9 (0.1)	7535
Coverage with artificial material	6220	4 (0.1)	4 (0.06)	9 (0.1)	7291
Parietal pleurectomy	22	0	0	0	27
Coverage and parietal pleurectomy	68	0	0	0	54
Others	118	0	0	0	163
Others	639	3 (0.5)	1 (0.2)	8 (1.3)	610
Unknown	2	0	0	0	9
Total	9592	8 (0.1)	8 (0.1)	23 (0.2)	10,924

Secondary pneumothorax					
Associated disease	Cases	30-Day mortality		Hospital mortality	VATS
		Hospital	After discharge		
COPD	2775	41 (1.5)	11 (0.4)	83 (3.0)	2644
Tumorous disease	173	5 (2.9)	4 (2.3)	10 (5.8)	160
Catamenial	178	0	0	0	176
LAM	37	0	0	0	37
Others (excluding pneumothorax by trauma)	759	13 (1.7)	4 (0.5)	30 (4.0)	706
Unknown	0	0	0	0	0

Operative procedure	Cases	30 Day mortality		Hospital mortality	VATS
		Hospital	After discharge		
Bullectomy	718	4 (0.6)	4 (0.6)	12 (1.7)	690
Bullectomy with additional procedure	2243	32 (1.4)	8 (0.4)	53 (2.4)	2174
Coverage with artificial material	2152	29 (1.3)	7 (0.3)	48 (2.2)	2088
Parietal pleurectomy	10	1 (10.0)	0	1 (10.0)	10
Coverage and parietal pleurectomy	23	0	0	2 (8.7)	22
Others	58	2 (3.4)	1 (1.7)	2 (3.4)	54
Others	959	23 (2.4)	7 (0.7)	58 (6.0)	857
Unknown	2	0	0	0	2
Total	3922	59 (1.5)	19 (0.5)	123 (3.1)	3723

(), Mortality %

The 2020 survey reported 180 procedures for chest wall deformity (Table 23). However, this may have been underestimated because the Nuss procedure for pectus excavatum was more likely performed in pediatric surgery centers not associated with the Japanese Association for Thoracic Surgery.

**Table 23:** F. Chest wall deformity

	Cases	30-Day mortality		Hospital mortality
		Hospital	After discharge	
F. Chest wall deformity	180	0	0	0
Funnel chest	168	0	0	0
Others	12	0	0	0

(), Mortality %

Surgical treatment for diaphragmatic hernia was performed in 41 patients (Table 24). This may have been underestimated because procedures may have been classified as gastrointestinal surgery.

**Table 24:** G. Diaphragmatic hernia

	Cases	30-Day mortality		Hospital mortality	VATS
		Hospital	After discharge		
G. Diaphragmatic hernia	41	0	0	1 (2.4)	21
Congenital	8	0	0	0	2
Traumatic	11	0	0	0	6
Others	22	0	0	1 (4.5)	13

(), Mortality %

The survey reported 458 procedures for chest trauma, excluding iatrogenic injuries (Table 25), with a hospital mortality rate of 6.6%.

**Table 25:** H. Chest trauma

	Cases	30-Day mortality		Hospital mortality	VATS
		Hospital	After discharge		
H. Chest trauma	458	26 (5.7)	0	30 (6.6)	253

(), Mortality %

Table 26 summarizes the procedures for other diseases, including 92 and 99 cases of arteriovenous malformation and pulmonary sequestration, respectively.

**Table 26:** I. Other respiratory surgery

	Cases	30-Day mortality		Hospital mortality	VATS
		Hospital	After discharge		
I. Other respiratory surgery	1899	54 (2.8)	8 (0.4)	95 (5.0)	1407
Arteriovenous malformation*	92	0	0	0	86
Pulmonary sequestration	99	0	0	0	83
Postoperative bleeding ·air leakage	541	21 (3.9)	4 (0.7)	37 (6.8)	362
Chylothorax	67	0	0	2 (3.0)	56
Others	1100	33 (3.0)	4 (0.4)	56 (5.1)	820

(), Mortality %

A total of 75 lung transplantations were performed in 2020 (Table 27), among which 58 and 17 were from brain-dead and living-related donors, respectively.

**Table 27:** Lung transplantation

	Cases	30-Day mortality		Hospital mortality
		Hospital	After discharge	
Single lung transplantation from brain-dead donor	31	0	0	0
Bilateral lung transplantation from brain-dead donor	27	1 (3.7)	0	1 (3.7)
Lung transplantation from living donor	17	2 (11.8)	0	3 (17.6)
Total lung transplantation	75	0	0	4 (5.3)
Donor of living donor lung transplantation	29	0	0	0

(), Mortality %

In 2020, the number of VATS procedures decreased by 1.2% from 77,059 to 76,073 compared to that of 2019 with the decrease of all procedures in general thoracic surgery (− 5.3%). However, the population of VATS procedures in all procedures increased to 88% in 2020 compared that in 2019 (84%) (Table 28).

**Table 28:** Video-assisted thoracic surgery

	Cases	30-Day mortality		Hospital mortality
		Hospital	After discharge	
11. Video-assisted thoracic surgery	76,073	242 (0.3)	79 (0.1)	469 (0.6)

(), Mortality % (including thoracic sympathectomy 330)

A total of 665 tracheobronchoplasty procedures were performed in 2020, including 401 sleeve lobectomies, 17 carinal reconstructions and 10 sleeve pneumonectomies (Table 29). 30-day mortality for sleeve lobectomy, carinal reconstruction and sleeve lobectomy were 2, 6 and 10% respectively.

**Table 29:** Tracheobronchoplasty

	Cases	30-Day mortality		Hospital mortality
		Hospital	After discharge	
12. Tracheobronchoplasty	665	5 (0.8)	4 (0.6)	16 (2.4)
Trachea	38	0	0	0
Sleeve resection with reconstruction	25	0	0	0
Wedge with simple closure	6	0	0	0
Wedge with patch closure	0	0	0	0
Total laryngectomy with tracheostomy	0	0	0	0
Others	7	0	0	0
Carinal reconstruction	17	0	0	1 (5.9)
Sleeve pneumonectomy	10	1 (10.0)	0	1 (10.0)
Sleeve lobectomy	401	2 (0.5)	0	8 (2.0)
Sleeve segmental excision	13	0	0	0
Bronchoplasty without lung resection	17	0	0	1 (5.9)
Others	169	2 (1.2)	4 (2.4)	5 (3.0)

(), Mortality %

Tables 30, 31, 32 present the details regarding pediatric surgery and combined resection of neighboring organs.

**Table 30:** Pediatric surgery

	Cases	30-Day mortality		Hospital mortality
		Hospital	After discharge	
13. Pediatric surgery	297	9 (3.0)	0	10 (3.4)

(), Mortality %

**Table 31:** Combined resection of neighboring organ(s)

	Cases	30-Day mortality		Hospital mortality
		Hospital	After discharge	
14. Combined resection of neighboring organ(s)	1300	7 (0.5)	0	16 (1.2)

Organ resected	Cases	30-Day mortality		Hospital mortality
		Hospital	After discharge	
A. Primary lung cancer				
Aorta	9	0	0	0
Superior vena cava	24	0	0	0
Brachiocephalic vein	7	0	0	1 (14.3)
Pericardium	63	2 (3.2)	0	2 (3.2)
Pulmonary artery	117	1 (0.9)	0	2 (1.7)
Left atrium	14	0	0	1 (7.1)
Diaphragm	63	0	0	2 (3.2)
Chest wall (including ribs)	276	3 (1.1)	0	8 (2.9)
Vertebra	10	0	0	0
Esophagus	3	0	0	0
Total	586	6 (1.0)	0	16 (2.7)
B. Mediastinal tumor				
Aorta	1	0	0	0
Superior vena cava	63	1 (1.6)	0	2 (3.2)
Brachiocephalic vein	130	0	0	0
Pericardium	364	0	0	0
Pulmonary artery	4	0	0	0
Left atrium	0	0	0	0
Diaphragm	43	0	0	0
Chest wall (including ribs)	9	0	0	0
Vertebra	7	0	0	0
Esophagus	8	0	0	0
Lung	524	0	0	0
Total	1153	1 (0.1)	0	2 (0.2)

(), Mortality %

**Table 32:** Operation of lung cancer invading the chest wall of the apex

	Cases	30-Day mortality		Hospital mortality
		Hospital	After discharge	
15. Operation of lung cancer invading the chest wall of the apex	655	3 (0.5)	0	7 (1.1)

(), Mortality %

Includes tumors invading the anterior apical chest wall and posterior apical chest wall (superior sulcus tumor, so called Pancoast type)

### (C) Esophageal surgery

In 2018, the data collection method for esophageal surgery had been modified from self-reports using questionnaire sheets following each institution belonging to the Japanese Association for Thoracic Surgery to an automatic package downloaded from the NCD in Japan. Consequently, the registry excluded data for non-surgical cases with esophageal diseases. Furthermore, data regarding the histological classification of malignant tumors, multiple primary cancers, and mortality rates for cases with combined resection of other organs could not be registered because they were not included in the NCD. Instead, detailed data regarding postoperative surgical and non-surgical complications were collected from the NCD. Moreover, data regarding surgeries for corrosive esophageal strictures and salvage surgeries for esophageal cancer had been exceptionally registered by participating institutions.

**Fig. 3:**
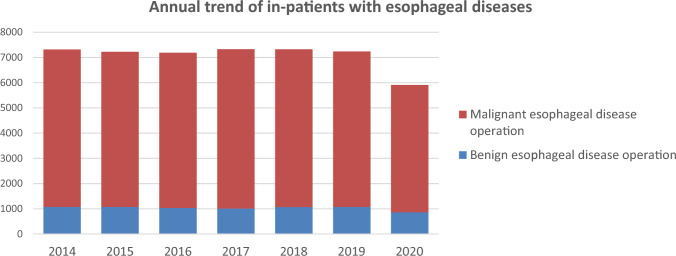
Annual trend of in-patients with esophageal diseases

Throughout 2020, 5909 patients underwent surgery for esophageal diseases (860 and 5049 for benign and malignant esophageal diseases, respectively) from institutions across Japan. Compared to 2019, there was a total decrease of 1326 cases (18.3%) observed, with a decrease of 214 cases (19.9%) in benign diseases and a decrease of 1112 cases (18.0%) in malignant diseases. It is considered that this significant decline was largely influenced by the COVID-19 pandemic that began in 2020, with factors such as surgical restrictions, reduced medical visits, and postponed screenings being considered as contributing factors (Fig. 3).

Concerning benign esophageal diseases (Table 33), thoracoscopic and/or laparoscopic surgeries were performed in 90.7% (68/75), 84.6% (357/422), 100% (27/27), and 36.7% (62/169) of patients with esophagitis (including esophageal ulcer), hiatal hernia, benign tumors, and achalasia, respectively. Conversely, 100% (92/92) of patients with spontaneous rupture of the esophagus underwent open surgery. Hospital mortality rates within 30 postoperative days were 0.5% (2/422), 4.3% (4/92) for hiatal hernia and spontaneous rupture of the esophagus, respectively.

**Table 33:** Benign esophageal diseases

	Operation (+)				T/L*3			
	Cases	Hospital mortality			Cases	Hospital mortality		
		~ 30 days	31–90 days	Total (including after 91 days mortality)		~ 30 days	31–90 days	Total (including after 91 days mortality)
1. Achalasia	169	0	0	0	62	0	0	0
2. Benign tumor	27	0	0	0	27	0	0	0
3. Diverticulum	28	0	0	0	5	0	0	0
4. Hiatal hernia	422	2 (0.5)	0	2 (0.5)	357	1 (0.3)		1 (0.3)
5. Spontaneous rupture of the esophagus	92	4 (4.3)	3 (3.3)	7 (7.6)	0	0	0	0
6. Esophago-tracheal fistula	3	0	0	0	0	0	0	0
7. Esophagitis, Esophageal ulcer	75	0	0	0	68	0	0	0
8. Corrosive stricture of the esophagus	44	0	0	0	17	0	0	0
Total	860	6 (0.7)	3 (0.3)	9 (1.0)	536	1 (0.2)	0	1 (0.2)

(), Mortality %

*T/L* thoracoscopic and/or laparoscopic

The most common tumor location for malignant esophageal diseases was the thoracic esophagus (Table 34). Among 5049 cases with esophageal malignancies, esophagectomy for superficial and advanced cancers was performed in 1927 (38.2%) and 3122 (61.8%), respectively. Hospital mortality rates within 30 days after esophagectomy were 0.6% and 0.5% for patients with superficial and advanced cancer, respectively.

**Table 34:** Malignant esophageal disease

	Operation (+)				Thoracoscopic and/or laparoscopic procedure				
	Cases	Hospital mortality			Cases	Conversion to thoracotomy	Hospital mortality		
		~ 30 days	31–90 days	Total (including after 91 days mortality)			~ 30 days	31–90 days	Total (including after 91 days mortality)
Location									
(1) Cervical esophagus	138				64				
(2) Thoracic esophagus	4222	23 (0.5)	13 (0.3)	36 (0.9)	3675	33 (0.9)	20 (0.5)	10 (0.3)	31 (0.8)
(3) Abdominal esophagus	410	1 (0.2)		1 (0.2)	331	2 (0.6)	1 (0.3)		1 (0.3)
Total	4770	24 (0.5)	13 (0.3)	37 (0.8)	4070	35 (0.9)	21 (0.5)	10 (0.2)	32 (0.8)
Tumor depth									
(A) Superficial cancer (T1)									
(1) Transhiatal esophagectomy	8								
(2) Mediastinoscopic esophagectomy and reconstruction	115				115				
(3) Transthoracic (rt.) esophagectomy and reconstruction	1221	9 (0.7)	4 (0.3)	13 (1.1)	1102	9 (0.8)	9 (0.8)	3 (0.3)	12 (1.1)
(4) Transthoracic (lt.) esophagectomy and reconstruction	25	1 (4.0)			14				
(5) Cervical esophageal resection and reconstruction	20								
(6) Robot-assisted esophagectomy and reconstruction	357	1 (0.3)		2 (0.6)	355	1 (0.3)	1 (0.3)		1 (0.3)
(7) Others	12								
(8) Esophagectomy without reconstruction	169				60				
Subtotal	1927	11 (0.6)	4 (0.2)	15 (0.8)	1646	10 (0.6)	10 (0.6)	3 (0.2)	13 (0.8)
(B) Advanced cancer (T2–T4)									
(1) Transhiatal esophagectomy	11								
(2) Mediastinoscopic esophagectomy and reconstruction	127		1 (0.8)	1 (0.8)	127			1 (0.8)	1 (0.8)
(3) Transthoracic (rt.) esophagectomy and reconstruction	2267	10 (0.4)	6 (0.3)	16 (0.7)	1836	23 (1.3)	8 (0.4)	5 (0.3)	13 (0.7)
(4) Transthoracic (lt.) esophagectomy and reconstruction	42	1 (2.4)	1 (2.4)	2 (4.8)	26		1 (3.8)		1 (3.8)
(5) Cervical esophageal resection and reconstruction	51								
(6) Robot-assisted esophagectomy and reconstruction	479	2 (0.4)	1 (0.2)	3 (0.6)	479	1 (0.2)	2 (0.4)	1 (0.2)	3 (0.6)
(7) Others	35								
(8) Esophagectomy without reconstruction	110	2 (1.8)	5 (4.5)	7 (6.4)					
Subtotal	3122	15 (0.5)	14 (0.4)	29 (0.9)	2468	24 (1.0)	11 (0.4)	7 (0.3)	18 (0.7)
Total	5049	26 (0.5)	18 (0.4)	44 (0.9)	4114	34 (0.8)	21 (0.5)	10 (0.2)	31 (0.8)

	Cases	Overall morbidity	Morbidity ≥ CD III	Surgical complications					
				Surgical site infection			Anastomotic leakage	Recurrent nerve palsy	Wound dehiscence
				Superficial incision	Deep incision	Organ space			
Location									
(1) Cervical esophagus	138	87 (63.0)	40 (29.0)	15 (10.9)	13 (9.4)	13 (9.4)	21 (15.2)	17 (12.3)	2 (1.4)
(2) Thoracic esophagus	4222	2406 (57.0)	954 (22.6)	322 (7.6)	172 (4.1)	371 (8.8)	573 (13.6)	622 (14.7)	69 (1.6)
(3) Abdominal esophagus	410	212 (51.7)	93 (22.7)	28 (6.8)	17 (4.1)	33 (8.0)	62 (15.1)	30 (7.3)	4 (1.0)
Total	4770	2705 (56.7)	1087 (22.8)	365 (7.7)	202 (4.2)	417 (8.7)	656 (13.8)	669 (14.0)	75 (1.6)
Tumor depth									
(A) Superficial cancer (T1)									
(1) Transhiatal esophagectomy	8	3 (37.5)	2 (25.0)	1 (12.5)				1 (12.5)	
(2) Mediastinoscopic esophagectomy and reconstruction	115	77 (67.0)	32 (27.8)	9 (7.8)	4 (3.5)	6 (5.2)	22 (19.1)	37 (32.2)	1 (0.9)
(3) Transthoracic (rt.) esophagectomy and reconstruction	1221	680 (55.7)	270 (22.1)	96 (7.9)	46 (3.8)	110 (9.0)	185 (15.2)	161 (13.2)	20 (1.6)
(4) Transthoracic (lt.) esophagectomy and reconstruction	25	13 (52.0)	6 (24.0)	1 (4.0)	2 (8.0)	2 (8.0)	3 (12.0)	1 (4.0)	
(5) Cervical esophageal resection and reconstruction	20	12 (60.0)	6 (30.0)	2 (10.0)	4 (20.0)	1 (5.0)	4 (20.0)	2 (10.0)	1 (5.0)
(6) Robot-assisted esophagectomy and reconstruction	357	190 (53.2)	72 (20.2)	27 (7.6)	15 (4.2)	31 (8.7)	46 (12.9)	42 (11.8)	3 (0.8)
(7) Others	12	4 (33.3)	1 (8.3)			1 (8.3)			0
(8) Esophagectomy without reconstruction	169								
Subtotal	1927	979 (50.8)	389 (20.2)	136 (7.1)	71 (3.7)	151 (7.8)	260 (13.5)	244 (12.7)	25 (1.3)
(B) Advanced cancer (T2–T4)									
(1) Transhiatal esophagectomy	11	7 (63.6)	4 (36.4)	1 (9.1)	1 (9.1)	2 (18.2)	3 (27.3)	2 (18.2)	
(2) Mediastinoscopic esophagectomy and reconstruction	127	82 (64.6)	30 (23.6)	13 (10.2)	10 (7.9)	15 (11.8)	22 (17.3)	22 (17.3)	3 (2.4)
(3) Transthoracic (rt.) esophagectomy and reconstruction	2267	1313 (57.9)	544 (24.0)	170 (7.5)	97 (4.3)	210 (9.3)	313 (13.8)	313 (13.8)	40 (1.8)
(4) Transthoracic (lt.) esophagectomy and reconstruction	42	20 (47.6)	3 (7.1)	3 (7.1)	1 (2.4)	4 (9.5)	5 (11.9)	2 (4.8)	0
(5) Cervical esophageal resection and reconstruction	51	33 (64.7)	7 (13.7)	7 (13.7)	4 (7.8)	3 (5.9)	3 (5.9)	10 (19.6)	0
(6) Robot-assisted esophagectomy and reconstruction	479	253 (52.8)	104 (21.7)	33 (6.9)	16 (3.3)	31 (6.5)	46 (9.6)	76 (15.9)	7 (1.5)
(7) Others	35	18 (51.4)	6 (17.1)	2 (5.7)	2 (5.7)	1 (2.9)	4 (11.4)		
(8) Esophagectomy without reconstruction	110								
Subtotal	3122	1726 (55.3)	698 (22.4)	229 (7.3)	131 (4.2)	266 (8.5)	396 (12.7)	425 (13.6)	50 (1.6)
Total	5049	2705 (53.6)	1087 (21.5)	365 (7.2)	202 (4.0)	417 (8.3)	656 (13.0)	669 (13.3)	75 (1.5)

	Cases	Nonsurgical complications									Readmission within 30 days	Reoperation within 30 days
		Pneumonia	Unplanned intubation	Prolonged ventilation > 48 h	Pulmonary embolism	Atelectasis	Renal failure	CNS events	Cardiac events	Septic shock		
Location												
(1) Cervical esophagus	138	14 (10.1)	8 (5.8)	12 (8.7)	1 (0.7)	1 (0.7)		1 (0.7)	1 (0.7)		3 (2.2)	21 (15.2)
(2) Thoracic esophagus	4222	637 (15.1)	165 (3.9)	192 (4.5)	51 (1.2)	204 (4.8)	20 (0.5)	15 (0.4)	20 (0.5)	29 (0.7)	114 (2.7)	279 (6.6)
(3) Abdominal esophagus	410	47 (11.5)	17 (4.1)	19 (4.6)	4 (1.0)	24 (5.9)		1 (0.2)	1 (0.2)	3 (0.7)	3 (0.7)	30 (7.3)
Total	4770	698 (14.6)	190 (4.0)	223 (4.7)	56 (1.2)	229 (4.8)	20 (0.4)	17 (0.4)	22 (0.5)	32 (0.7)	120 (2.5)	330 (6.9)
Tumor depth												
(A) Superficial cancer (T1)												
(1) Transhiatal esophagectomy	8	1 (12.5)										1 (12.5)
(2) Mediastinoscopic esophagectomy and reconstruction	115	14 (12.2)	4 (3.5)	5 (4.3)		6 (5.2)			4 (3.5)		4 (3.5)	7 (6.1)
(3) Transthoracic (rt.) esophagectomy and reconstruction	1221	173 (14.2)	52 (4.3)	63 (5.2)	11 (0.9)	39 (3.2)	7 (0.6)	6 (0.5)	5 (0.4)	10 (0.8)	25 (2.0)	79 (6.5)
(4) Transthoracic (lt.) esophagectomy and reconstruction	25	3 (12.0)	2 (8.0)	2 (8.0)		5 (20.0)					1 (4.0)	
(5) Cervical esophageal resection and reconstruction	20	4 (20.0)	2 (10.0)			1 (5.0)						2 (10.0)
(6) Robot-assisted esophagectomy and reconstruction	357	41 (11.5)	6 (1.7)	10 (2.8)	6 (1.7)	20 (5.6)	1 (0.3)			1 (0.3)	5 (1.4)	21 (5.9)
(7) Others	12	1 (8.3)				1 (8.3)						
(8) Esophagectomy without reconstruction	169											
Subtotal	1927	237 (12.3)	66 (3.4)	80 (4.2)	17 (0.9)	72 (3.7)	8 (0.4)	6 (0.3)	9 (0.5)	11 (0.6)	35 (1.8)	110 (5.7)
(B) Advanced cancer (T2–T4)												
(1) Transhiatal esophagectomy	11	1 (9.1)									2 (18.2)	
(2) Mediastinoscopic esophagectomy and reconstruction	127	18 (14.2)	6 (4.7)	5 (3.9)	1 (0.8)	7 (5.5)	1 (0.8)		1 (0.8)	2 (1.6)	5 (3.9)	9 (7.1)
(3) Transthoracic (rt.) esophagectomy and reconstruction	2267	361 (15.9)	99 (4.4)	116 (5.1)	30 (1.3)	125 (5.5)	9 (0.4)	9 (0.4)	10 (0.4)	12 (0.5)	71 (3.1)	168 (7.4)
(4) Transthoracic (lt.) esophagectomy and reconstruction	42	4 (9.5)	2 (4.8)	2 (4.8)	1 (2.4)	2 (4.8)	1 (2.4)			2 (4.8)		2 (4.8)
(5) Cervical esophageal resection and reconstruction	51	3 (5.9)	1 (2.0)	2 (3.9)		1 (2.0)		1 (2.0)			1 (2.0)	5 (9.8)
(6) Robot-assisted esophagectomy and reconstruction	479	69 (14.4)	14 (2.9)	16 (3.3)	7 (1.5)	22 (4.6)	1 (0.2)	1 (0.2)	2 (0.4)	5 (1.0)	6 (1.3)	35 (7.3)
(7) Others	35	4 (11.4)	2 (5.7)	2 (5.7)		1 (2.9)			1 (2.9)			1 (2.9)
(8) Esophagectomy without reconstruction	110											
Subtotal	3122	460 (14.7)	124 (4.0)	143 (4.6)	39 (1.2)	158 (5.1)	12 (0.4)	11 (0.4)	14 (0.4)	21 (0.7)	85 (2.7)	220 (7.0)
Total	5049	697 (13.8)	190 (3.8)	223 (4.4)	56 (1.1)	230 (4.6)	20 (0.4)	17 (0.3)	23 (0.5)	32 (0.6)	120 (2.4)	330 (6.5)

**Fig. 4:**
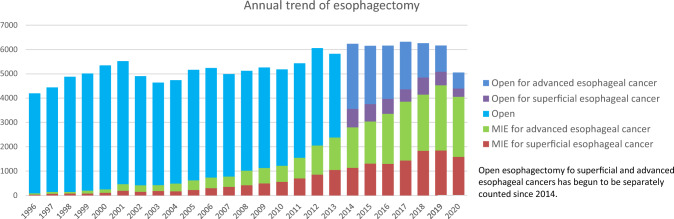
Annual trend of esophagectomy

Among esophagectomy procedures, transthoracic esophagectomy via right thoracotomy or right thoracoscopy was most commonly adopted for patients with superficial (1221/1927, 63.7%) and advanced cancer (2267/3122, 72.6%) (Table 34). Transhiatal esophagectomy, which is commonly performed in Western countries, was adopted in only 8 (0.4%) and 11 (0.4%) patients with superficial and advanced cancer who underwent esophagectomy in Japan, respectively. Thoracoscopic and/or laparoscopic esophagectomy was utilized in 1646 (85.4%) and 2468 (79.0%) patients with superficial and advanced cancer, respectively. Incidence of thoracoscopic and/or laparoscopic surgery (minimally invasive esophagectomy: MIE) for superficial or advanced cancer have been increasing, whereas that of open surgery, especially for advanced cancer, has been decreasing annually (Fig. 4). Mediastinoscopic esophagectomy was slightly increased, and performed for 115 (6.0%) and 127 (4.1%) patients with superficial and advanced esophageal cancer, respectively. Robot-assisted esophagectomy has been remarkably increased since 2018 when the insurance approval was obtained in Japan, and performed for 355 (18.4%) and 479 (15.3%) patients with superficial and advanced esophageal cancer, respectively in 2020. Patients who underwent robot-assisted surgery are increasing for both superficial and advancer esophageal cancers compared to that in 2019 (12.3% and 9.9% in 2019, respectively). Hospital mortality rates within 30 days after thoracoscopic and/or laparoscopic esophagectomy were 0.6% and 0.4% for patients with superficial and advanced cancer, respectively (Table 34).

Detailed data collection regarding postoperative surgical and non-surgical complications was initiated in 2018. Overall, 1087 (21.5%) of 5049 patients developed grade III or higher complications based on the Clavien–Dindo classification in 2020 (Table 34). The incidence of grade III or higher complications was relatively higher in cervical esophageal cancer compared to thoracic or abdominal esophageal cancer. Among surgical complications, anastomotic leakage and recurrent nerve palsy occurred in 14.2% and 13.6% of the patients who underwent right transthoracic esophagectomy, in 11.0% and 14.1% of those who underwent robot-assisted esophagectomy, and in 18.2% and 24.4% of those who underwent mediastinoscopic esophagectomy, respectively. Among non-surgical postoperative complications, pneumonia occurred in 13.8% of the patients, 3.8% of whom underwent unplanned intubation. Postoperative pulmonary embolism occurred in 1.1% of the patients. These complication rates, including the others, were similar to those in 2019.

Salvage surgery following definitive (chemo)radiotherapy was safely performed in 300 patients in 2020, with hospital mortality rates of 0% within 30 days postoperatively. (Table 35).

**Table 35:** Salvage surgery

	Operation (+)				Thoracoscopic and/or laparoscopic procedure					EMR or ESD
	Cases	Hospital mortality			Cases	Conversion to thoracotomy	Hospital mortality			
		~ 30 days	31–90 days	Total (including after 91 days mortality)			~ 30 days	31–90 days	Total (including after 91 days mortality)	
Salvage surgery	300	0	2 (0.7)	2 (0.7)	188	4 (2.1)	0	0	0	89

We aim to continue our efforts in collecting comprehensive survey data through more active collaboration with the Japan Esophageal Society and other related institutions, with caution due to the impact of COVID-19 pandemic.

## Data Availability

Based on the data use policy of JATS, data access is approved through assessment by the JATS: Committee for Scientific Affairs. Those interested in using the data should contact the JATS: Committee for Scientific Affairs (survey@jpats.org) to submit a proposal. The use of the data is granted for the approved study proposals.
