# Correction: Thoracic and cardiovascular surgeries in Japan during 2020: Annual report by the Japanese Association for Thoracic Surgery

**DOI:** 10.1007/s11748-024-02112-z

**Published:** 2025-01-03

**Authors:** Committee for Scientific Affairs, The Japanese Association for Thoracic Surgery, Goro Matsumiya, Yukio Sato, Hiroya Takeuchi, Tomonobu Abe, Shunsuke Endo, Yasutaka Hirata, Michiko Ishida, Hisashi Iwata, Takashi Kamei, Nobuyoshi Kawaharada, Shunsuke Kawamoto, Kohji Kohno, Hiraku Kumamaru, Kenji Minatoya, Noboru Motomura, Rie Nakahara, Morihito Okada, Hisashi Saji, Aya Saito, Hideyuki Shimizu, Kenji Suzuki, Hirofumi Takemura, Tsuyoshi Taketani, Yasushi Toh, Wataru Tatsuishi, Hiroyuki Yamamoto, Takushi Yasuda, Masayuki Watanabe, Naoki Yoshimura, Masanori Tsuchida, Yoshiki Sawa

**Affiliations:** 1 The Japanese Association for Thoracic Surgery, Committee for Scientific Affairs, Tokyo, Japan; 2 Department of Cardiovascular Surgery, Chiba University Graduate School of Medicine, Chiba, Japan; 3 Department of Thoracic Surgery, University of Tsukuba, Tsukuba, Japan; 4 Department of Surgery, Hamamatsu University School of Medicine, Shizuoka, Japan; 5 Division of Cardiovascular Surgery, Department of General Surgical Science, Gunma University, Maebashi, Japan; 6 Thoracic Surgery, Jichi Medical University Saitama Medical Center, Omiya, Japan; 7 Department of Cardiac Surgery, The University of Tokyo Hospital, Tokyo, Japan; 8 Cardiac Surgery, Handa City Hospita, Aichi, Japan; 9 Department of General Thoracic Surgery, Gifu University Hospital, Gifu, Japan; 10 Department of Surgery, Graduate School of Medicine, Tohoku University, Sendai, Japan; 11 Department of Cardiovascular Surgery, Sapporo Medical University School of Medicine, Sapporo, Japan; 12 Department of Cardiovascular Surgery, Tohoku Medical and Pharmaceutical University Hospital, Sendai, Japan; 13 Department of Gastrointestinal Tract Surgery, Fukushima Medical University, Fukushima, Japan; 14 Department of Healthcare Quality Assessment, Graduate School of Medicine, The University of Tokyo, Tokyo, Kagoshima Japan; 15 Department of Cardiovascular Surgery, Graduate School of Medicine, Kyoto University, Kyoto, Japan; 16 Department of Cardiovascular Surgery, Toho University Sakura Medical Center, Chiba, Japan; 17 Division of Thoracic Surgery, Tochigi Cancer Center, Toshigi, Japan; 18 Surgical Oncology, Hiroshima University, Hiroshima, Japan; 19 Department of Chest Surgery, St. Marianna University School of Medicine, Kawasaki, Japan; 20 Department of Surgery, Yokohama City University, Graduate School of Medicine, Yokohama, Japan; 21 Department of Cardiovascular Surgery, Keio University, Tokyo, Japan; 22 Department of General Thoracic Surgery, Juntendo University School of Medicine, Tokyo, Japan; 23 Department of Cardiovascular Surgery, Kanazawa University, Kanazawa, Japan; 24 Department of Cardiovascular Surgery, Mitsui Memorial Hospital, Tokyo, Japan; 25 Department of Gastroenterological Surgery, National Hospital Organization Kyushu Cancer Center, Fukuoka, Japan; 26 Department of Surgery, Kindai University Faculty of Medicine, Osaka, Japan; 27 Department of Gastroenterological Surgery, Cancer Institute Hospital, Tokyo, Japan; 28 Department of Thoracic and Cardiovascular Surgery, University of Toyama, Graduate School of Medicine, Toyama, Japan; 29 Division of Thoracic and Cardiovascular Surgery, Niigata University Graduate School of Medical and Dental Sciences, Niigata, Japan; 30 Osaka University, Graduate School of Medicine, Osaka Police Hospital, Osaka, Japan

**Correction: General Thoracic and Cardiovascular Surgery (2024) 72:61–94** 10.1007/s11748-023-01979-8

In the original publication of the article [1], the number of 30-day hospital mortality and 30-day hospital mortality rate for the Stanford type A acute aortic dissections in “Table 3 (1) Dissection” were published incorrectly. The corrected part of the table is given in this Correction. The corrected number of 30-day hospital mortality was 493, and hospital mortality rate was 8.0%, respectively.

**Table 3:** Thoracic aortic aneurysm (total; 22,540) (1) Dissection (total; 10,855)

Stanford type	Acute								Chronic								Concomitant operation					
	A				B				A				B									
Replaced site	Cases	30-day mortality		Hospital mortality	Cases	30-day mortality		Hospital mortality	Cases	30-day mortality		Hospital mortality	Cases	30-day mortality		Hospital mortality	AVP	AVR	MVP	MVR	CABG	Others
		Hospital	After discharge			Hospital	After discharge			Hospital	After discharge			Hospital	After discharge							
Ascending Ao	2071	146 (7.0)	1 (0.05)	189 (9.1)	1	0	0	0	187	6 (3.2)	0	8 (4.3)	1	0	0	0	61	137	22	8	110	32
Aortic Root	191	35 (18.3)	0	36 (18.8)	0	0	0	0	80	4 (5.0)	0	5 (6.3)	3	0	0	0	32	194	6	2	65	7
Arch	1954	135 (6.9)	1 (0.05)	174 (8.9)	31	0	0	0	355	9 (2.5)	0	13 (3.7)	172	5 (2.9)	0	6 (3.5)	54	113	10	5	118	25
Aortic root + asc. Ao. + Arch	167	23 (13.8)	0	26 (15.6)	0	0	0	0	47	1 (2.1)	0	3 (6.4)	6	0	0	0	23	143	2	0	35	2
Descending Ao	35	4 (11.4)	0	4 (11.4)	28	2 (7.1)	0	2 (7.1)	56	1 (1.8)	0	1 (1.8)	220	9 (4.1)	0	10 (4.5)	2	4	0	0	4	0
Thoracoabdominal	1	1 (100.0)	0	1 (100.0)	11	1 (9.1)	0	1 (9.1)	46	5 (10.9)	0	5 (10.9)	182	11 (6.0)	1 (0.5)	13 (7.1)	0	0	0	0	1	0
Simple TEVAR	101	9 (8.9)	0	11 (10.9)	442	30 (6.8)	0	34 (7.7)	264	2 (0.8)	0	3 (1.1)	1171	7 (0.6)	0	8 (0.7)	1	2	0	0	2	2
Open SG with BR	1213	101 (8.3)	2 (0.16)	133 (11.0)	62	3 (4.8)	0	3 (4.8)	207	8 (3.9)	0	11 (5.3)	237	4 (1.7)	0	7 (3.0)	61	115	10	2	104	16
Open SG without BR	435	32 (7.4)	0	45 (10.3)	28	2 (7.1)	0	3 (10.7)	52	2 (3.8)	0	4 (7.7)	82	3 (3.7)	0	3 (3.7)	20	45	1	0	30	2
Arch TEVAR with BR	14	1 (7.1)	0	1 (7.1)	123	6 (4.9)	0	10 (8.1)	73	2 (2.7)	0	2 (2.7)	364	7 (1.9)	0	8 (2.2)	1	0	0	0	0	0
Thoracoabdominal TEVAR with BR	0	0	0	0	11	0	0	0	6	0	0	1 (16.7)	33	2 (6.1)	0	4 (12.1)	0	0	0	0	0	0
Other	18	6 (33.3)	0	6 (33.3)	13	3 (23.1)	0	3 (23.1)	10	0	0	0	51	2 (3.9)	0	2 (3.9)	0	2	0	0	1	1
Total	6200	493 (8.0)	4 (0.06)	626 (10.1)	750	47 (6.3)	0	56 (7.5)	1383	40 (2.9)	0	56 (4.0)	2522	50 (2.0)	1 (0.0)	61 (2.4)	255	755	51	17	470	87

(), % mortality

*Ao* aorta, *AVP* aortic valve repair, *AVR* aortic valve replacement, *MVP* mitral valve repair, *MVR* mitral valve replacement, *CABG* coronary artery bypass grafting, *TEVAR* thoracic endovascular aortic (aneurysm) repair

Acute, within 2 weeks from the onset
